# Comparison of Internal Validation Strategies for Estimating Machine Learning Model Performance in One-Year Mortality Prediction in Hepatocellular Carcinoma

**DOI:** 10.3390/jcm15207899

**Published:** 2026-10-13

**Authors:** Zeynep Kucukakcali, Sami Akbulut, Kemal Baris Sarici

**Affiliations:** 1 Department of Biostatistics and Medical Informatics, Faculty of Medicine, Inonu University, 44280 Malatya, Türkiye; zeynep.tunc@inonu.edu.tr; 2 Department of Surgery and Liver Transplantation, Faculty of Medicine, Inonu University, 44280 Malatya, Türkiye; kemal.sarici@inonu.edu.tr

**Keywords:** hepatocellular carcinoma, one-year mortality prediction, clinical prediction model, machine learning, internal validation, nested cross-validation, calibration, decision curve analysis, SHAP

## Abstract

**Background**: Machine learning (ML) performance estimates in hepatocellular carcinoma (HCC) may depend on internal validation strategy, especially in small datasets. **Aim**: To develop models for one-year mortality prediction and compare performance, calibration, decision-analytic performance, and uncertainty estimates using a conventional train–test split and nested cross-validation (nCV). **Methods**: We analyzed a public dataset of 165 patients with HCC, including 63 one-year deaths. Random Forest (RF), XGBoost, and CatBoost were developed using fold-specific preprocessing, cost-sensitive learning, Optuna-based hyperparameter optimization, and training-based threshold selection. L2-penalized logistic regression provided a conventional benchmark. Models were evaluated using a stratified 80/20 split and five-fold nCV with five inner folds. Six stratified splits assessed partition variability, and unweighted models assessed the effect of class weighting. Discrimination, threshold-dependent classification, calibration, and decision-analytic performance were assessed using ROC-AUC, PR-AUC, balanced accuracy, Matthew’s correlation coefficient, Brier score, calibration curves, and decision-curve analysis. Confidence intervals used 2000 bootstrap resamples; SHAP was used for interpretation. **Results**: In the conventional split, RF and CatBoost achieved the highest test-set ROC-AUC (0.892 each), while RF achieved the highest PR-AUC (0.860). Under nCV, RF had the highest mean outer-fold ROC-AUC (0.788) and pooled out-of-fold ROC-AUC (0.784). XGBoost had the highest mean PR-AUC (0.673), whereas CatBoost had the highest pooled PR-AUC (0.648). RF had the lowest mean Brier score (0.188), followed by XGBoost (0.194) and CatBoost (0.203). Calibration varied across models, with pooled calibration slopes of 1.438, 0.691, and 0.427, respectively. Across six 80/20 splits, mean ROC-AUC values were 0.792, 0.769, and 0.761 for RF, XGBoost, and CatBoost, respectively, with substantial partition-dependent variation. Removing class weighting yielded broadly comparable performance. Bootstrap confidence intervals were narrower for pooled nCV predictions, but the evaluation samples differed in size (165 versus 33 patients), precluding attribution of this difference to validation strategy alone. Decision-curve analysis was exploratory because no clinically actionable threshold was prespecified. SHAP identified AFP, ALP, hemoglobin, albumin, performance status, and AST among the most influential RF predictors; the highest-ranked predictors were generally consistent across outer folds. **Conclusions**: nCV separated model development from evaluation and reduced reliance on a single small test set. RF was selected by the prespecified mean outer-fold ROC-AUC criterion, but differences among tree-based models were small and no clear discrimination advantage over penalized logistic regression was demonstrated. External validation is needed before clinical use.

## 1. Introduction

Hepatocellular carcinoma (HCC) is the predominant histological subtype of primary liver cancer and remains a major cause of cancer-related morbidity and mortality worldwide [1,2]. Although chronic hepatitis B virus (HBV) and hepatitis C virus (HCV) infections remain major etiological drivers, the epidemiological profile of HCC is changing, with increasing contributions from alcohol-associated liver disease and metabolic dysfunction-associated steatotic liver disease [1,2]. These epidemiological and healthcare challenges reinforce the importance of accurate prognostic assessment to support individualized management of patients with HCC [3,4].

Prognostic assessment in HCC is inherently complex because outcomes reflect the combined influence of tumor burden, underlying liver function, performance status, comorbidities, and treatment-related factors. The Barcelona Clinic Liver Cancer (BCLC) classification provides an established framework for staging and therapeutic decision-making; however, it may not fully capture the heterogeneity in individual patient characteristics and tumor behavior that contributes to variation in prognosis [3,4,5]. Individualized prognostic estimation is therefore relevant to treatment planning and risk stratification. However, translating multiple demographic, clinical, laboratory, and tumor-related characteristics into patient-level prognostic estimates remains methodologically challenging [6,7].

Artificial intelligence encompasses computational approaches to tasks such as pattern recognition and decision support, while machine learning (ML) enables models to learn from data and generate predictions. In healthcare, these approaches have been investigated using routinely collected clinical data to support diagnostic assessment and predict patient outcomes [8,9]. Oncology studies have applied ML to prognostic questions, including the prediction of survival and cancer recurrence. These applications are particularly relevant to HCC, where prognosis reflects the combined influence of tumor characteristics, liver function, and patient-related factors. ML methods have therefore been investigated for HCC prognostic modeling because they can represent nonlinear relationships and interactions among clinical variables [10]. Recent studies illustrate the breadth of these applications across different HCC populations and outcomes. Dong et al. [11] evaluated six ML algorithms for 1-, 3-, and 5-year survival prediction in 2038 patients with alpha-fetoprotein (AFP)- positive HCC. Jie Shen et al. [12] compared Cox regression, deep learning, and ML approaches in patients with advanced multiple HCC, whereas Yang et al. [5] evaluated eight ML algorithms for overall survival after curative hepatectomy in large HCC and incorporated model interpretation using SHapley Additive exPlanations (SHAP). Collectively, these studies show both the expanding role and methodological diversity of ML-based HCC prognostication.

This methodological diversity extends to the structure and evaluation of HCC prognostic models. Using longitudinal time-series data from 2511 patients with HCC, Shen et al. [13] compared Survival Path and Dynamic DeepHit and showed that relative predictive performance varied according to prediction and evaluation time. Zhao et al. [14] evaluated Cox-based methods, random survival forests, deep learning models, and ensemble approaches using Surveillance, Epidemiology, and End Results (SEER) and The Cancer Genome Atlas (TCGA) data. Validation procedures also vary among HCC studies. For example, Dong et al. [11] used a 7:3 training–test partition with cross-validation within the training set for model tuning, while other studies employed different internal or external evaluation frameworks. Thus, reported performance across HCC studies is generated under heterogeneous analytical conditions, complicating direct comparisons and making it difficult to attribute observed differences solely to algorithmic capability [4,5,6,7,11,12,13,14].

How a model is developed and evaluated is therefore an important component of interpreting predictive performance. A systematic review by Andaur Navarro et al. [15] identified frequent risk-of-bias concerns in supervised ML prediction-model studies, including issues related to sample size, missing data, and assessment of overfitting. Contemporary methodological guidance likewise emphasizes transparent reporting and appropriate separation between model development and evaluation. TRIPOD+AI provides updated reporting guidance for clinical prediction models using regression or ML methods [16], while PROBAST+AI distinguishes model development from model evaluation when assessing methodological quality, risk of bias, and applicability [17].

Internal validation strategy is central to this framework. A single random train–test split is straightforward but may yield performance estimates that depend substantially on which observations are assigned to the development and test samples, particularly when sample size is limited. An et al. [18] demonstrated substantial variation in estimated performance across repeated random train–test partitions in small-sample prediction settings. Nested cross-validation (nCV) provides an alternative framework in which model-development procedures, including hyperparameter optimization, are performed within inner resampling loops and predictive performance is evaluated on previously unseen outer folds. Methodological studies indicate that this separation can limit information leakage and model-selection optimism [19,20]. Importantly, nCV does not eliminate uncertainty arising from limited sample size; rather, it provides a structured approach for separating model optimization from internal performance evaluation.

These considerations are directly relevant to HCC prognostic ML research. Although recent studies have evaluated a broad range of algorithms and prognostic settings, their populations, outcomes, model-development procedures, data-partitioning schemes, and validation approaches differ substantially [4,5,6,7,11,12,13,14]. Consequently, it remains difficult to determine whether differences in reported predictive performance primarily reflect algorithmic capability or the analytical framework used for model development and evaluation. A controlled comparison in which preprocessing, class-imbalance handling, hyperparameter optimization, threshold selection, and performance evaluation are kept constant while the validation framework is varied may therefore help clarify the contribution of validation design to estimated model performance and its uncertainty.

Accordingly, the present study developed Random Forest (RF), Extreme Gradient Boosting (XGBoost), and Categorical Boosting (CatBoost) models to predict one-year mortality using the publicly available University of California, Irvine (UCI) HCC Survival dataset. An identical analytical pipeline was applied across models, including preprocessing, class-imbalance handling, hyperparameter optimization, threshold selection, and performance evaluation. A conventional stratified 80/20 train–test split was compared with five-fold nCV with respect to discrimination, threshold-dependent classification performance, calibration, clinical utility, and uncertainty of performance estimates. The primary objective was to examine how internal validation strategy influences estimated model performance and its uncertainty when the surrounding analytical pipeline is otherwise held constant.

## 2. Materials and Methods

### 2.1. Study Design

This methodological prediction-modeling study was based on a secondary analysis of a publicly available retrospective clinical dataset. The study aimed to develop and internally validate ML models for predicting one-year mortality in patients with HCC. Three tree-based ensemble algorithms were compared using an identical analytical pipeline, and the influence of the validation strategy on predictive performance, calibration, uncertainty, clinical utility, and variability in performance estimates was evaluated.

Two validation frameworks were examined: a conventional stratified 80/20 train–test split and five-fold nCV. nCV was prespecified as the primary validation framework because it separates model optimization from performance estimation and reduces the risk of optimism associated with model selection. The single train–test split was retained as a secondary conventional comparison, while variability across train–test partitions was examined separately using six stratified splits. Accordingly, model selection and the primary interpretation of predictive performance were based on the nCV framework, whereas the single train–test split served as a complementary comparison of validation-dependent performance estimates.

### 2.2. Dataset

The publicly available HCC Survival dataset was obtained from the UCI Machine Learning Repository [21]. The dataset was originally collected by the Internal Medicine A Service of the Coimbra Hospital and University Centre in Portugal and comprises clinical data from 165 HCC patients [22]. The variables include demographic characteristics, behavioral and etiological risk factors, comorbidities, manifestations of chronic liver disease, tumor-related characteristics, laboratory measurements, and one-year survival status. The original dataset developers selected the clinical variables with reference to the European Association for the Study of the Liver–European Organisation for Research and Treatment of Cancer (EASL–EORTC) Clinical Practice Guidelines for the management of HCC [23].

The dataset comprises 49 candidate predictor variables and one binary outcome variable. According to the accompanying data dictionary, the predictors include 23 quantitative and 26 qualitative variables, reflecting the heterogeneous clinical structure of the dataset. Candidate predictors encompassed age at diagnosis, sex, alcohol, grams of alcohol per day, smoking, packs of cigarettes per year, hepatitis B surface antigen (HBsAg), hepatitis B e antigen (HBeAg), hepatitis B core antibody (Anti-HBc), and hepatitis C virus antibody, cirrhosis, diabetes, obesity, arterial hypertension, chronic renal insufficiency, human immunodeficiency virus (HIV), nonalcoholic steatohepatitis (NASH), hemochromatosis, portal hypertension (PHT), esophageal varices, splenomegaly, portal vein thrombosis (PVT), ascites degree, encephalopathy degree, performance status, number of hepatic nodules, maximum diameter of the largest nodule, radiologic hallmark of HCC, symptoms at presentation, residence in an endemic country or region and routinely available laboratory measurements.

Laboratory variables included alpha-fetoprotein (AFP), international normalized ratio (INR), total and direct bilirubin, alanine aminotransferase (ALT), aspartate aminotransferase (AST), alkaline phosphatase (ALP), gamma-glutamyl transferase (GGT), hemoglobin, mean corpuscular volume (MCV), leukocyte count, platelet count, ferritin, iron, creatinine, albumin, total protein, and oxygen saturation. In the original dataset, 826 of 8085 predictor values were missing (10.22%), and only eight patients (4.85%) had complete data for all 49 candidate predictors.

Before model development, seven of the 49 candidate predictors—liver metastasis, grams of alcohol per day, packs of cigarettes per year, direct bilirubin, iron, oxygen saturation, and ferritin—were excluded, leaving 42 predictors for modeling. These seven predictors were not used in model training, hyperparameter optimization, or performance evaluation. Among the 42 retained predictors, 438 of 6930 values were missing (6.32%), and 31 patients (18.8%) had complete data for all retained predictors. Excluding these predictors did not exclude any patients; all 165 patients were retained in the modeling analyses. Missing values in the retained predictors were handled within the model-development pipeline, as described below. Predictor-specific missingness is presented in Supplementary Table S1.

This secondary analysis used an existing, publicly available dataset; therefore, the sample size was fixed, and no formal a priori sample-size calculation was performed. The small number of patients (*n* = 165) relative to the 42 retained predictors was considered when designing the internal validation strategy and interpreting model performance.

### 2.3. Outcome Definition

The original dataset defined the target outcome as one-year survival, coded as 1 for survival and 0 for death within one year. For the present analysis, the outcome was recoded so that death within one year after diagnosis represented the positive class (event = 1), whereas survival at one year represented the negative class (event = 0). This transformation changed only the orientation of the binary outcome and did not alter the underlying event distribution. The recoding ensured that sensitivity, positive predictive value, and related classification measures referred directly to one-year mortality. Among the 165 patients, 63 died within one year and 102 survived, corresponding to a one-year mortality prevalence of 38.2% and a one-year survival proportion of 61.8%. Accordingly, one-year mortality constituted the minority class, although the class distribution was not extremely imbalanced. The observed class distribution was considered during model training, classification-threshold selection, and performance evaluation.

### 2.4. Data Preprocessing

Predictor variables were categorized as binary, ordinal, or continuous according to the original data dictionary. Binary and ordinal predictors were retained using their original numerical coding. Continuous variables were analyzed on their original scales because the evaluated tree-based algorithms did not require standardization or normalization. Values denoted by question marks in the original data file were converted to standard missing values before preprocessing. Missing observations were handled within a unified scikit-learn pipeline to prevent information leakage. Binary and ordinal variables were imputed using the most frequent value in the relevant training partition, whereas continuous variables were imputed using the median of the corresponding training partition.

All preprocessing parameters were estimated exclusively from the data available for model training within each validation iteration and were subsequently applied to the corresponding validation or test observations. In the conventional train–test analysis, preprocessing was fitted only within the 80% training sample. In nCV, preprocessing was refitted within the relevant training partitions. No patient was excluded solely because of missing predictor values; therefore, all 165 patients were retained in the modeling analyses.

Because one-year mortality constituted the minority class (38.2%), cost-sensitive learning was used to account for class imbalance without generating synthetic observations in this small and clinically heterogeneous dataset. Given the relatively modest degree of class imbalance, an additional sensitivity analysis was performed without class weighting to assess whether the main findings were dependent on this modeling choice. RF models were trained using class_weight = “balanced”. For XGBoost, scale_pos_weight was calculated as the ratio of negative to positive observations in the relevant training partition. CatBoost models were trained using auto_class_weights = “Balanced”. Class weights were recalculated independently within each training partition and were not derived from the complete dataset. Consequently, the class distribution of the held-out test set or an outer test fold did not contribute to model fitting.

### 2.5. ML Models

Three tree-based ensemble classifiers were evaluated: RF, XGBoost, and CatBoost. These algorithms were selected because they can model nonlinear relationships and higher-order interactions without requiring prespecified functional forms. They are also suitable for heterogeneous tabular clinical data and can be interpreted using SHapley Additive exPlanations. RF constructs an ensemble of decision trees using bootstrap samples and random subsets of predictors, with final probabilities obtained by aggregating predictions across trees. XGBoost sequentially builds gradient-boosted decision trees while incorporating regularization and subsampling to control model complexity. CatBoost is a gradient-boosting implementation that incorporates ordered boosting and regularization procedures. The same preprocessing, missing-data management, class-imbalance strategy, hyperparameter optimization, threshold-selection procedure, and validation framework were applied to all three algorithms. Thus, the surrounding analytical framework was kept as consistent as possible across the three algorithms, facilitating comparison of their predictive performance. In addition, L2-penalized logistic regression was included as a conventional statistical benchmark to assess whether the tree-based ML approaches provided incremental predictive performance over a simpler linear modeling approach. The penalized logistic regression model was evaluated using the same predictor set and nCV framework, with preprocessing restricted to the corresponding training partitions and regularization strength optimized within the inner cross-validation loop. For penalized logistic regression, continuous predictors were standardized within each training partition, and the resulting scaling parameters were applied to the corresponding validation or outer-fold test observations. This provided a conventional benchmark evaluated using the same predictors and nested cross-validation framework, with model-specific preprocessing fitted within the training partitions.

### 2.6. Hyperparameter Optimization

Model hyperparameters were optimized using the Optuna framework with a Tree-structured Parzen Estimator sampler. Algorithm-specific search spaces were defined before model evaluation. For RF, the search space included the number of trees, maximum tree depth, minimum number of observations required for node splitting and terminal nodes, number of predictors considered at each split, and bootstrap sampling. For XGBoost, the search space included the number of boosting iterations, maximum tree depth, learning rate, row and column subsampling ratios, minimum child weight, gamma, and L1 and L2 regularization parameters. For CatBoost, the search space included the number of boosting iterations, tree depth, learning rate, L2 regularization, random-strength parameters, and subsampling-related settings.

For each model and each optimization process, 15 Optuna trials were conducted. The optimization objective was the mean area under the receiver operating characteristic curve obtained from the relevant inner five-fold cross-validation procedure. Hyperparameter tuning was restricted entirely to training data. No hyperparameter was selected by examining the held-out test set or an outer test fold. The number of trials was selected as a pragmatic balance between the breadth of hyperparameter exploration and computational burden, as hyperparameter optimization was repeatedly performed within the inner loops of the nested cross-validation framework.

Predicted probabilities were converted into binary classifications using a data-driven threshold rather than a fixed threshold of 0.50. Within each training sample, out-of-fold probabilities were generated through inner five-fold cross-validation. Candidate thresholds were evaluated using these training-only predictions, and the threshold maximizing balanced accuracy was selected.

The selected threshold was then fixed and applied without modification to the corresponding unseen test observations. In nCV, threshold optimization was repeated independently within each outer training fold. In the conventional 80/20 split, the threshold was determined exclusively from cross-validated predictions generated within the 80% training sample. This procedure-maintained separation between threshold selection and final performance evaluation.

### 2.7. Validation Strategies

The same modeling pipeline was evaluated under two validation frameworks. Five-fold nCV was used as the primary validation strategy, whereas a conventional stratified 80/20 train–test split was used as a secondary comparison.

#### 2.7.1. Conventional Train–Test Split

For the initial conventional split analysis, the complete cohort was divided into an 80% training set and a 20% held-out test set using stratified random sampling with a random seed of 42. Stratification preserved the proportion of one-year deaths and survivors in both partitions. The resulting test set contained 33 patients. All preprocessing, class-weight calculation, hyperparameter optimization, and classification-threshold selection were conducted exclusively within the training set. Hyperparameter optimization and threshold selection were based on stratified five-fold cross-validation within the 80% training sample.

After the optimal model configuration had been identified, the complete pipeline was refitted using all observations in the training set and evaluated once on the previously unseen test set. The test set was not used for imputation, class-weight estimation, hyperparameter selection, threshold selection, or model comparison. Because performance estimates depended on the composition of one randomly selected and relatively small test sample, results from this framework were considered secondary.

#### 2.7.2. nCV

Five-fold nCV was used as the primary internal-validation framework. The nested procedure consisted of an outer loop for estimating generalization performance and an inner loop for model development. Both outer and inner folds were stratified according to the outcome. At each outer iteration, four folds were assigned to model development and the remaining fold was reserved as an unseen outer test set. Within the outer training sample, inner five-fold cross-validation was used for preprocessing, class-weight estimation, hyperparameter optimization, and classification-threshold selection.

After the optimal hyperparameters and threshold had been determined, the complete model pipeline was refitted using the entire outer training sample and evaluated on the corresponding outer test fold. This process was repeated across all five outer folds. Each patient was therefore included in an outer test fold exactly once and received one prediction from a model that had not been trained, tuned, or optimized using that patient.

The outer-fold predictions were concatenated to create a complete set of out-of-fold predictions for all 165 patients. These pooled predictions were used to estimate cohort-level discrimination, threshold-dependent classification performance, calibration, and clinical net benefit. Fold-specific metrics were additionally summarized using means and standard deviations to describe between-fold variability. By restricting hyperparameter optimization and classification-threshold selection to the inner loop, nCV separated these model-development steps from performance estimation in the outer folds. A fixed random seed of 42 was applied to data splitting, model fitting, and optimization procedures where supported.

### 2.8. Performance Evaluation

Model performance was evaluated across four complementary domains: discrimination, threshold-dependent classification performance, probability calibration, and decision-analytic performance. Discrimination was quantified using the area under the receiver operating characteristic curve and the area under the precision–recall curve, reported as average precision. ROC-AUC reflected the probability that a randomly selected patient who died within one year received a higher predicted mortality probability than a randomly selected survivor. PR-AUC was included because it provides additional information regarding model performance in the positive class when outcome classes are unequally distributed. The observed one-year mortality prevalence of 38.2% represented the no-skill reference level for the precision–recall analysis.

Threshold-dependent classification performance was evaluated using balanced accuracy, overall accuracy, F1 score, Matthew’s correlation coefficient, sensitivity, specificity, positive predictive value, and negative predictive value. Sensitivity represented the proportion of patients who died within one year and were correctly classified as high risk, whereas specificity represented the proportion of one-year survivors correctly classified as non-events. Balanced accuracy was calculated as the mean of sensitivity and specificity. Matthew’s correlation coefficient was included because it incorporates all four cells of the confusion matrix and remains informative in the presence of unequal class distributions.

Probability calibration was assessed using the Brier score and graphical calibration curves. The Brier score was calculated as the mean squared difference between predicted mortality probabilities and observed outcomes, with lower values indicating better overall probabilistic accuracy. Calibration curves compared predicted probabilities with observed event frequencies. Calibration intercept and calibration slope were additionally estimated from the pooled out-of-fold predictions. Ideal calibration corresponds to an intercept of 0 and a slope of 1.

Decision curve analysis was used to estimate net benefit across the evaluated range of threshold probabilities and to compare the models with two default strategies: classifying all patients as likely to die within one year and classifying no patients as likely to die. The findings were interpreted as an exploratory assessment across hypothetical threshold probabilities and not as evidence of established clinical utility or clinical effectiveness. For nCV, cohort-level metrics were calculated from pooled out-of-fold predictions, and fold-specific results were additionally summarized as means and standard deviations. For the conventional validation strategy, performance metrics were calculated from predictions generated for the held-out 20% test set.

### 2.9. Model Selection and SHAP Methodology

The model demonstrating the strongest overall performance under the primary nCV framework was selected as the final prediction model. The prespecified primary criterion for final model selection was mean outer-fold ROC-AUC. PR-AUC, calibration, threshold-dependent performance, and between-fold variability were considered as secondary performance characteristics. Results from the conventional 80/20 split were not used to select the final model. Following model selection, the selected algorithm was reoptimized using internal cross-validation across the complete dataset and then fitted using all 165 patients. This full-data model was developed for interpretation and potential future external validation. Its fitted performance was not treated as an independent estimate of generalization performance.

Model interpretability was investigated using SHapley Additive exPlanations. Global predictor importance was quantified using the mean absolute SHAP value across patients. Predictors were ranked according to their average absolute contribution to the model output. SHAP summary plots were generated to visualize both the magnitude and direction of each predictor’s contribution to one-year mortality predictions. Positive SHAP values indicated contributions toward a higher predicted probability of one-year mortality, whereas negative SHAP values indicated contributions toward a lower predicted probability. SHAP findings were interpreted as descriptions of the fitted model rather than estimates of causal effects or independent prognostic associations. Accordingly, high SHAP importance indicated that a variable contributed substantially to model predictions but did not establish that modifying that variable would alter clinical outcome.

To assess the stability of predictor importance, SHAP values were additionally calculated using each fold-specific RF model and the corresponding held-out outer-fold observations. For each predictor, stability was summarized using the mean absolute SHAP value across the five outer folds, mean rank, rank standard deviation, median rank, rank range, and the frequency of appearance among the top 5 and top 10 predictors.

### 2.10. Study Protocol, and Ethical Considerations

The study was conducted in accordance with the ethical principles of the Declaration of Helsinki, including its 2024 revision, and with applicable institutional and national regulations governing research involving human participants [24]. The present study involved no direct participant recruitment, intervention, or access to identifiable personal information, and all analyses were performed using a publicly available anonymized dataset. Therefore, additional informed consent was not required. Ethical approval was obtained from the Inonu University Scientific Research Ethics Committee for Health Sciences (session date: 25 August 2026; session number: 18; decision number: 11016). Reporting followed the TRIPOD+AI statement (Transparent Reporting of a multivariable prediction model for Individual Prognosis Or Diagnosis), an updated reporting guideline for clinical prediction models using regression or ML methods [16].

### 2.11. Statistical Analysis

Descriptive and comparative analyses of baseline clinical and laboratory characteristics according to one-year survival status were performed using IBM SPSS Statistics for Windows, version 25.0 (IBM Corp., Armonk, NY, USA). Continuous variables were summarized as median and interquartile range and compared between groups using the Mann–Whitney U test. Categorical variables were presented as counts and percentages and compared using Pearson’s chi-square test or an exact test, as appropriate according to expected cell frequencies.

Ninety-five percent confidence intervals were estimated using nonparametric percentile bootstrap resampling with 2000 iterations. For nCV, patients and their corresponding pooled out-of-fold predictions were resampled. For the conventional train–test analysis, bootstrap resampling was restricted to patients and predictions in the held-out test set. Bootstrap samples containing only one outcome class were excluded from the calculation of metrics requiring both classes. Confidence limits were defined by the 2.5th and 97.5th percentiles of the corresponding bootstrap distributions. Confidence-interval width was calculated as the difference between the upper and lower limits and was reported descriptively. Because the pooled nCV estimates were based on out-of-fold predictions from all 165 patients, whereas the conventional estimates were based on predictions from a 33-patient test set, interval widths were not interpreted as a direct comparison of the inherent precision of the two validation strategies.

The bootstrap confidence intervals were calculated from the available test set or out-of-fold predictions and therefore quantified uncertainty around the corresponding performance estimates; the complete model-development process was not repeated within each bootstrap sample. ML analyses were performed using Python (version 3.13.16) with scikit-learn (version 1.6.1), XGBoost (version 3.4.1), CatBoost (version 1.2.8), Optuna (version 4.4.0), and SHAP (version 0.52.0). Additional data processing and visualization were performed using NumPy (version 2.1.3), pandas (version 2.2.3), and Matplotlib (version 3.10.0). Reproducibility was supported through a fixed random seed of 42 and by embedding preprocessing, model fitting, threshold selection, and validation procedures within unified analytical pipelines.

## 3. Results

### 3.1. Cohort Characteristics

A total of 165 patients with HCC were included in the analysis. Following outcome recoding, 63 patients (38.2%) died within one year of diagnosis, whereas 102 patients (61.8%) survived at one year. Thus, one-year mortality constituted the minority class. All patients were retained in the modeling analyses because missing predictor values were handled using fold-specific imputation within the ML pipeline.

Table 1 presents the baseline continuous clinical and laboratory characteristics according to one-year survival status. Patients who died within one year were older and had higher median INR, AFP, platelet count, total bilirubin, AST, GGT, ALP, and maximum diameter of the largest nodule, whereas hemoglobin and albumin levels were lower (all *p* < 0.05). Table 2 presents differences in categorical clinical characteristics according to one-year survival status. Symptoms at presentation were more frequent among patients who died within one year than among one-year survivors (80.6% vs. 51.8%, *p* = 0.001). Portal vein thrombosis was more frequent among patients who died within one year (33.3% vs. 15.2%, *p* = 0.012). Performance-status distributions differed between the groups (*p* < 0.001), with a lower proportion of active patients among those who died within one year (27.0% vs. 61.8%). Ascites severity also differed according to one-year survival status (*p* = 0.004), as did hepatic encephalopathy grade (*p* = 0.049). These baseline comparisons were intended to characterize the study cohort rather than to identify independent prognostic factors or to perform predictor selection. All 42 predictors retained for model development were subsequently evaluated within the ML pipeline.

### 3.2. Performance of the Conventional 80/20 Train–Test Split

Table 3 shows the test-set performance of the three ML models under the conventional stratified 80/20 train–test split. In this analysis, 132 patients were used for model development and 33 patients constituted the held-out test set. RF and CatBoost achieved the highest ROC-AUC, both at 0.892, whereas RF achieved the highest PR-AUC at 0.860. RF had a balanced accuracy of 0.825, overall accuracy of 0.788, F1 score of 0.788, and Matthew’s correlation coefficient of 0.650. Its sensitivity was 1.000 and specificity was 0.650, with positive and negative predictive values of 0.650 and 1.000, respectively. The corresponding Brier score was 0.144.

XGBoost achieved an ROC-AUC of 0.862 and a PR-AUC of 0.755. Its balanced accuracy was 0.875, overall accuracy was 0.849, F1 score was 0.839, and Matthew’s correlation coefficient was 0.736. Sensitivity was 1.000 and specificity was 0.750, with positive and negative predictive values of 0.722 and 1.000, respectively. XGBoost achieved the lowest Brier score among the three models at 0.131.

CatBoost achieved an ROC-AUC of 0.892 and a PR-AUC of 0.791. Its threshold-dependent performance was identical to that of XGBoost for balanced accuracy (0.875), overall accuracy (0.849), F1 score (0.839), Matthew’s correlation coefficient (0.736), sensitivity (1.000), specificity (0.750), positive predictive value (0.722), and negative predictive value (1.000). Its Brier score was 0.159. Overall, no single model was uniformly superior across all test-set performance measures. RF and CatBoost achieved the highest ROC-AUC, RF achieved the highest PR-AUC, and XGBoost achieved the lowest Brier score. These single-split results were interpreted as secondary estimates rather than as the basis for final model selection. Supplementary Figures S1–S4 present ROC, precision–recall, calibration, and decision curves for the conventional 80/20 test set, respectively, while Supplementary Figures S5 and S6 present the corresponding SHAP analyses of the RF model.

#### Variability Across Stratified 80/20 Train–Test Splits

To assess the sensitivity of the conventional train–test validation strategy to data partitioning, the stratified 80/20 analysis was repeated using five additional random seeds (7, 19, 63, 88, and 123) in addition to the original seed of 42. The complete model-development procedure was repeated independently for each partition, including preprocessing, class-imbalance handling, five-fold internal cross-validation, Optuna-based hyperparameter optimization, and training-based threshold selection. Table 4 presents the performance metrics obtained for each individual random seed, together with the mean ± standard deviation (SD) and minimum–maximum values across the six stratified 80/20 partitions.

As shown in Table 4, performance estimates varied considerably across individual train–test partitions. RF ROC-AUC values ranged from 0.731 to 0.892, with a mean of 0.792 ± 0.061. XGBoost ROC-AUC values ranged from 0.658 to 0.862, with a mean of 0.769 ± 0.076, whereas CatBoost ROC-AUC values ranged from 0.673 to 0.892, with a mean of 0.761 ± 0.085. Partition-dependent variation was also observed for PR-AUC, Brier score, balanced accuracy, and MCC. The original seed-42 partition produced performance estimates toward the upper end of the observed ranges for all three models. Overall, these findings demonstrate that performance estimates derived from a single 80/20 train–test split can be substantially influenced by the specific allocation of patients to the training and test sets.

### 3.3. Primary Results of nCV

Table 5 shows the mean outer-fold performance of the three ML models obtained from five-fold nCV. RF achieved the highest mean ROC-AUC at 0.788 ± 0.064, followed by CatBoost at 0.784 ± 0.085 and XGBoost at 0.773 ± 0.042. XGBoost achieved the highest mean PR-AUC at 0.673 ± 0.018, followed closely by RF at 0.671 ± 0.070 and CatBoost at 0.669 ± 0.121. Mean balanced accuracy was 0.698 ± 0.076 for RF, 0.694 ± 0.104 for CatBoost, and 0.686 ± 0.039 for XGBoost. Mean Brier scores were 0.188 ± 0.027 for RF, 0.194 ± 0.035 for XGBoost, and 0.203 ± 0.053 for CatBoost.

**Figure 1:**
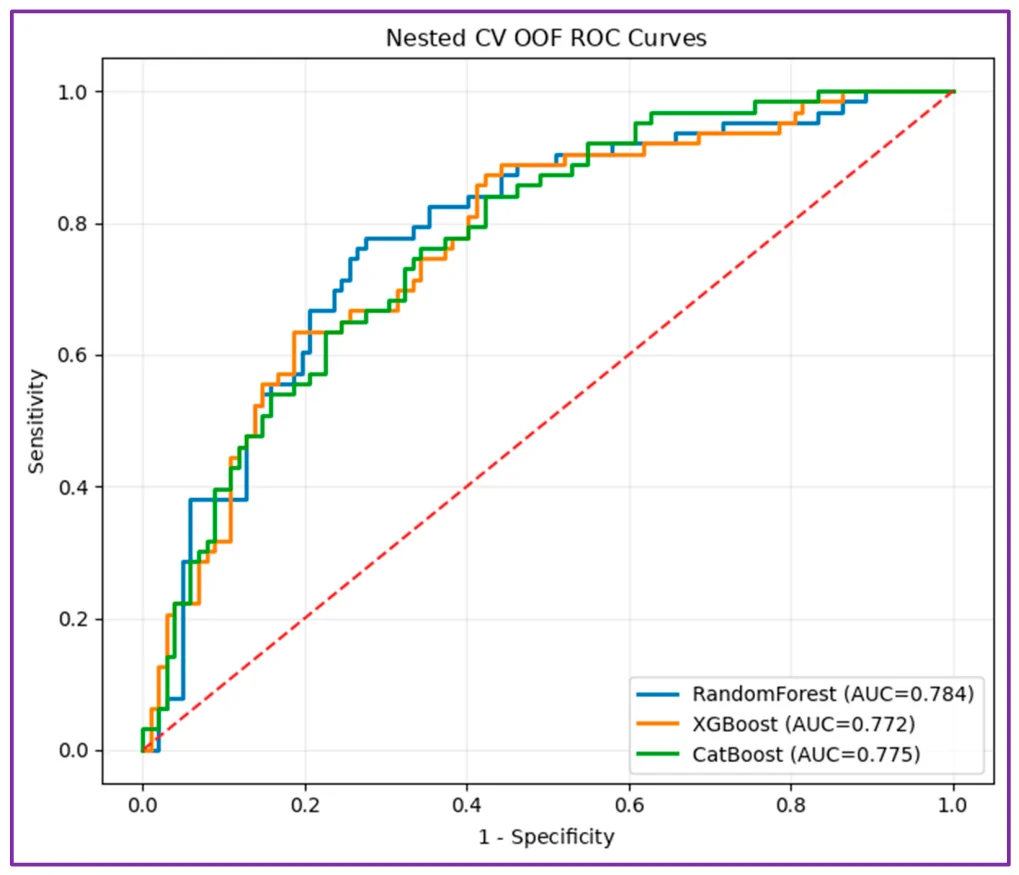
Receiver operating characteristic curves based on pooled out-of-fold predictions from nCV.

**Figure 2:**
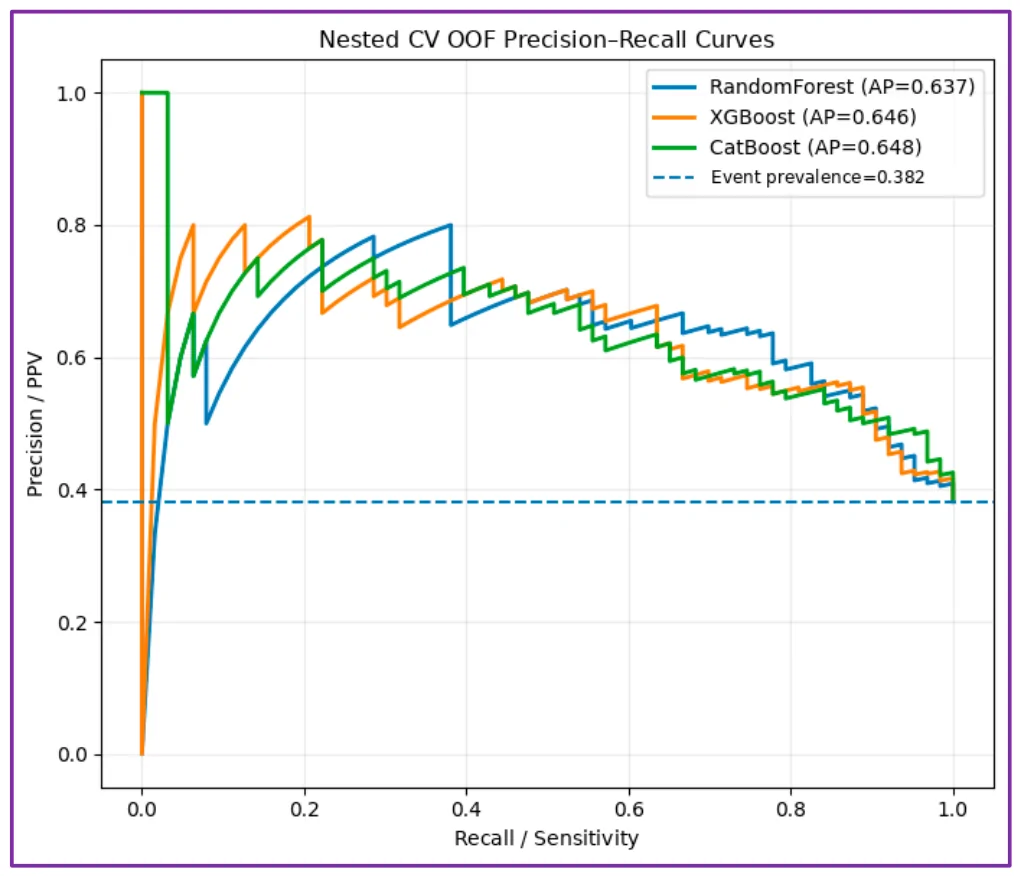
Precision–recall curves based on pooled out-of-fold predictions from nCV.

**Figure 3:**
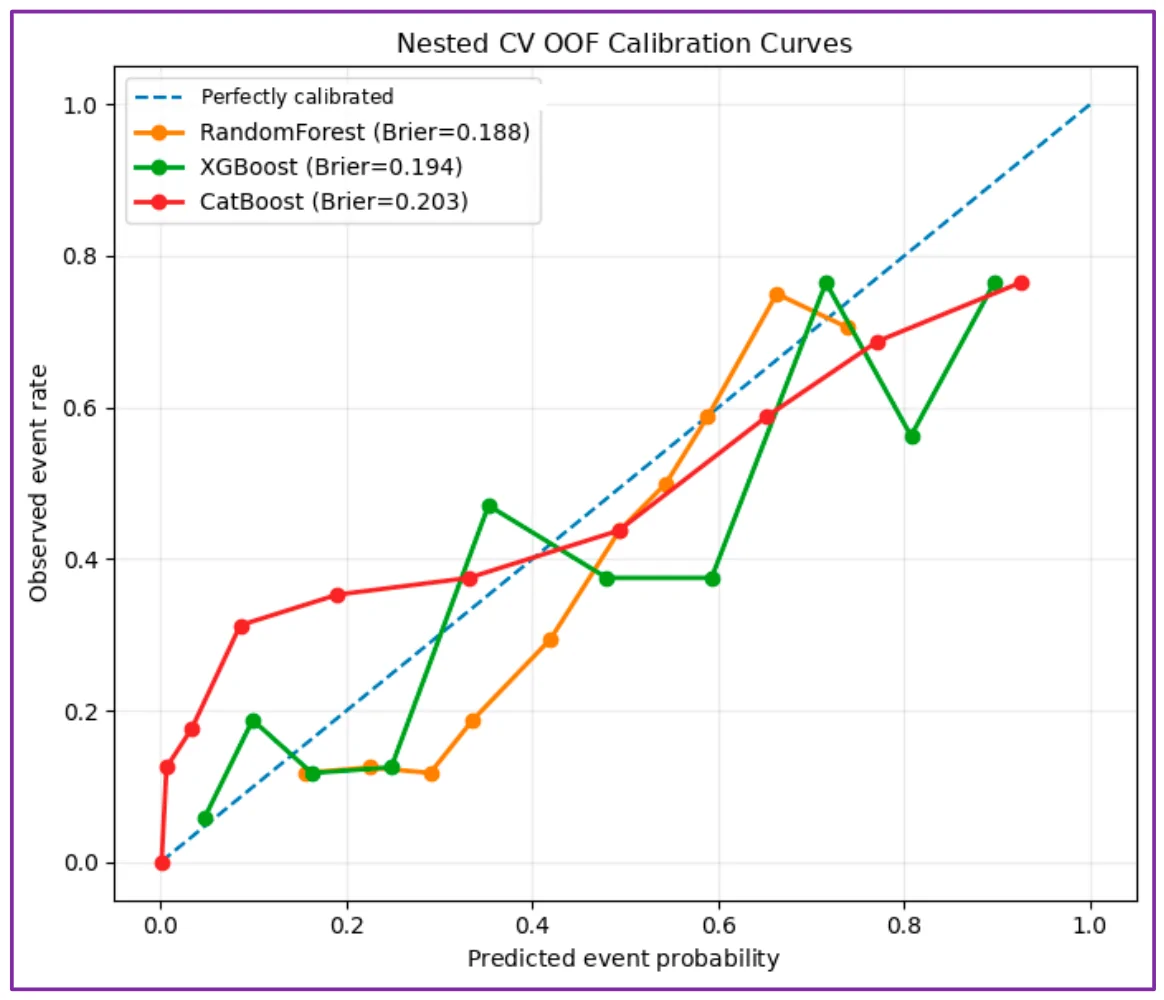
Calibration curves based on pooled out-of-fold predictions from nCV.

Pooled out-of-fold predictions were subsequently used to generate cohort-level ROC, precision–recall, calibration, and decision curves. When these predictions were analyzed, RF achieved the highest pooled ROC-AUC at 0.784, followed by CatBoost at 0.775 and XGBoost at 0.772. The ROC curves showed broadly similar discrimination across the three models (Figure 1). Precision–recall analysis likewise demonstrated similar performance across the models. Pooled PR-AUC values were 0.648 for CatBoost, 0.646 for XGBoost, and 0.637 for RF. All three values were above the no-skill reference corresponding to the observed one-year mortality prevalence of 0.382 (Figure 2). RF achieved the lowest pooled Brier score at 0.188, followed by XGBoost at 0.194 and CatBoost at 0.203. The calibration curves showed model-dependent deviations from ideal calibration, particularly at intermediate and higher predicted probabilities (Figure 3). To complement the visual assessment in Figure 3, calibration was also evaluated quantitatively using the pooled out-of-fold predictions. The calibration intercepts were −0.270, −0.355, and −0.002 for RF, XGBoost, and CatBoost, respectively, while the corresponding calibration slopes were 1.438, 0.691, and 0.427. The Brier scores were 0.188, 0.194, and 0.203, respectively. These findings indicate deviations from ideal calibration (intercept = 0 and slope = 1), particularly for the calibration slopes of XGBoost and CatBoost. Therefore, rather than indicating uniformly close agreement between predicted and observed risks, the calibration results suggest model-dependent miscalibration and should be interpreted cautiously, particularly given the limited sample size and the irregularity observed at intermediate and higher predicted probabilities.

**Figure 4:**
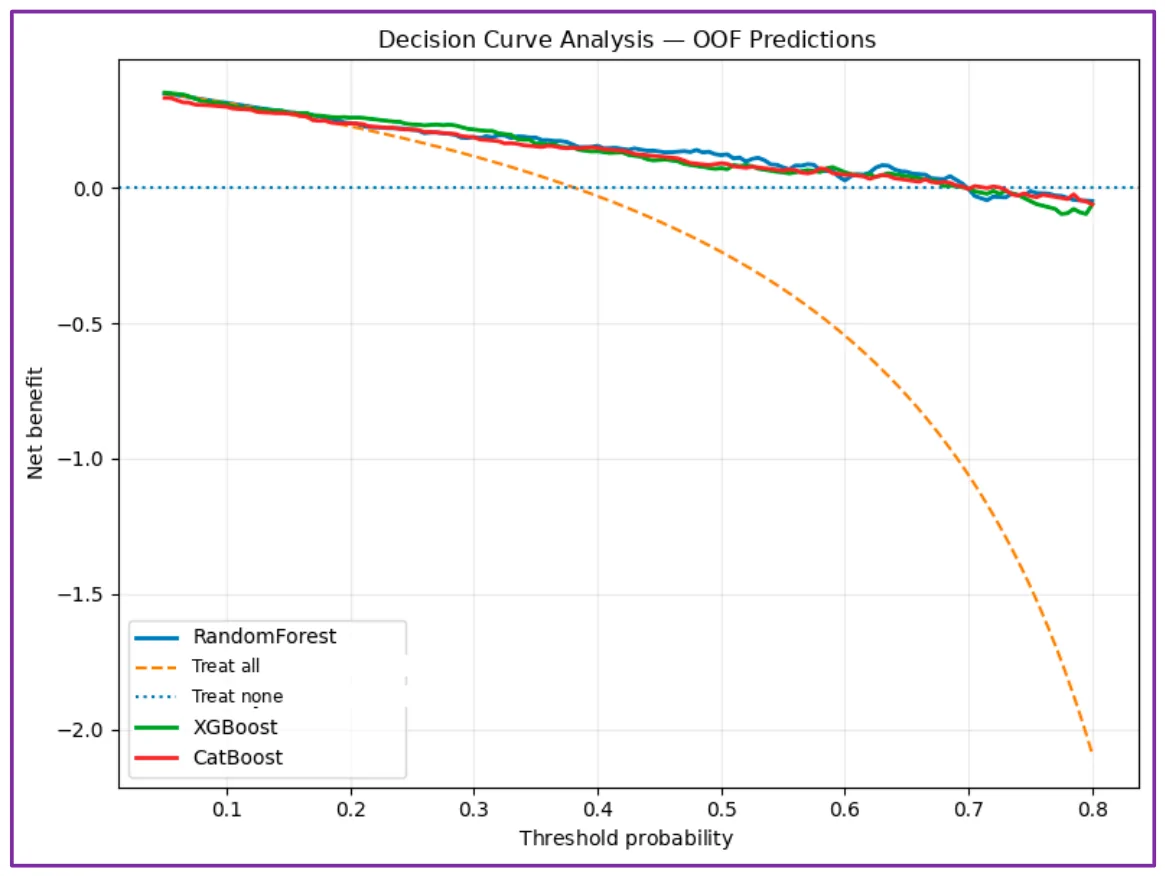
Decision curve analysis based on pooled out-of-fold predictions from nCV.

Decision curve analysis was performed as an exploratory assessment across the evaluated threshold-probability range (Figure 4). The three models showed broadly similar net-benefit profiles and achieved greater net benefit than the treat-all and treat-none strategies over portions of this range. However, the magnitude of net benefit was generally modest and became less stable at higher threshold probabilities. Because no clinically actionable threshold probability was prespecified for one-year mortality in this dataset, these findings should not be interpreted as evidence of established clinical utility. Rather, the decision curves provide an exploratory assessment of model performance across hypothetical risk thresholds; clinically meaningful thresholds should be prospectively defined according to a specific HCC decision context and subsequently evaluated in an independent cohort.

### 3.4. Comparison of Validation Strategies and Confidence Intervals

Table 6 presents the bootstrap 95% confidence intervals and corresponding interval widths for the primary performance metrics obtained using pooled nCV predictions and the conventional single train–test split. The two validation approaches yielded different point estimates and confidence-interval widths. The conventional train–test split generally produced higher point estimates, whereas the pooled nCV estimates showed narrower bootstrap confidence intervals.

For RF, the ROC-AUC obtained from the conventional test set was 0.892 (95% confidence interval [CI], 0.776–0.979), with an interval width of 0.203, whereas the pooled nCV ROC-AUC was 0.784 (95% CI, 0.711–0.851), with an interval width of 0.140. For XGBoost, the pooled nCV ROC-AUC was 0.772 (95% CI, 0.699–0.841), compared with 0.862 (95% CI, 0.722–0.975) in the conventional test set, with corresponding interval widths of 0.142 and 0.253, respectively. For CatBoost, the pooled nCV ROC-AUC was 0.775 (95% CI, 0.701–0.840), whereas the conventional test-set estimate was 0.892 (95% CI, 0.765–0.988), with corresponding interval widths of 0.139 and 0.223. Similar differences in confidence-interval widths were observed for PR-AUC and Brier score across the three algorithms.

Across all models, the mean ROC-AUC confidence-interval width was approximately 0.140 for the pooled nCV estimates and 0.226 for the conventional test-set estimates. However, these interval widths were derived from substantially different effective evaluation sample sizes. The pooled nCV estimates were based on out-of-fold predictions from all 165 patients, whereas the conventional estimates were based on a single test set of 33 patients. Therefore, the narrower confidence intervals observed for pooled nCV should not be interpreted as evidence of inherently greater precision of the nCV strategy, as the larger effective evaluation sample likely contributed to the observed difference in interval widths.

The confidence intervals for the three models also overlapped substantially, particularly for the conventional train–test estimates, indicating uncertainty in model performance estimates and model ranking. No formal pairwise hypothesis testing between models was performed. Accordingly, the confidence-interval comparison in Table 6 is presented descriptively to characterize the uncertainty associated with the performance estimates under each validation framework rather than to establish superior precision of one validation strategy over the other.

#### Sensitivity Analysis of Class Weighting

To assess whether the use of cost-sensitive learning materially influenced model performance, the complete nested cross-validation procedure with five outer folds and five inner folds was repeated without class weighting. The preprocessing, hyperparameter optimization, inner cross-validation, and threshold-selection procedures were kept consistent with the primary analysis, with class weighting being the only modeling component removed.

As shown in Table 7, the unweighted models demonstrated performance broadly comparable to that observed in the primary weighted analysis. Mean outer-fold ROC-AUC values were 0.808 ± 0.076 for RF, 0.780 ± 0.059 for XGBoost, and 0.805 ± 0.088 for CatBoost. The corresponding mean Brier scores were 0.184 ± 0.022, 0.192 ± 0.028, and 0.190 ± 0.035, respectively. Compared with the weighted analysis, the changes in discrimination and Brier scores were modest across all three algorithms.

The pooled out-of-fold results similarly showed no substantial deterioration after removal of class weighting. Calibration changes were model-dependent: the RF calibration slope increased from 1.438 to 1.571, whereas the slopes for XGBoost and CatBoost changed from 0.691 to 0.748 and from 0.427 to 0.574, respectively. Thus, although class weighting affected the estimated probabilities and calibration parameters to some extent, the sensitivity analysis did not indicate that the principal discrimination or overall prediction-error findings were materially dependent on the use of class weighting.

### 3.5. Model Selection for Interpretation

The predictive performance of RF, XGBoost, and CatBoost was broadly comparable under nCV. The mean outer-fold ROC-AUC values were 0.788 ± 0.064 for RF, 0.784 ± 0.085 for CatBoost, and 0.773 ± 0.042 for XGBoost. Similarly, the mean PR-AUC values were 0.671 ± 0.070, 0.669 ± 0.121, and 0.673 ± 0.018, respectively. RF yielded a pooled out-of-fold ROC-AUC of 0.784 and a mean Brier score of 0.188 ± 0.027, with mean sensitivity and specificity of 0.715 ± 0.086 and 0.680 ± 0.205, respectively.

Given the small differences in predictive performance among the three algorithms, these results do not provide evidence of a clear superiority of any single model. RF was selected for the subsequent interpretability analysis because it had the highest mean outer-fold ROC-AUC according to the prespecified primary selection criterion. This selection should therefore be regarded as a procedural choice for subsequent model interpretation rather than evidence of validated superiority over XGBoost or CatBoost.

For the subsequent SHAP analysis, RF was reoptimized using internal cross-validation across the complete dataset and fitted using all 165 patients. The performance of this full-data fitted model was not used as an independent estimate of generalization performance. The robustness of the resulting predictor-importance findings was additionally assessed across the five outer folds of nCV, as described in Section 3.6 and Supplementary Table S2.

### 3.6. Model Interpretation

**Figure 5:**
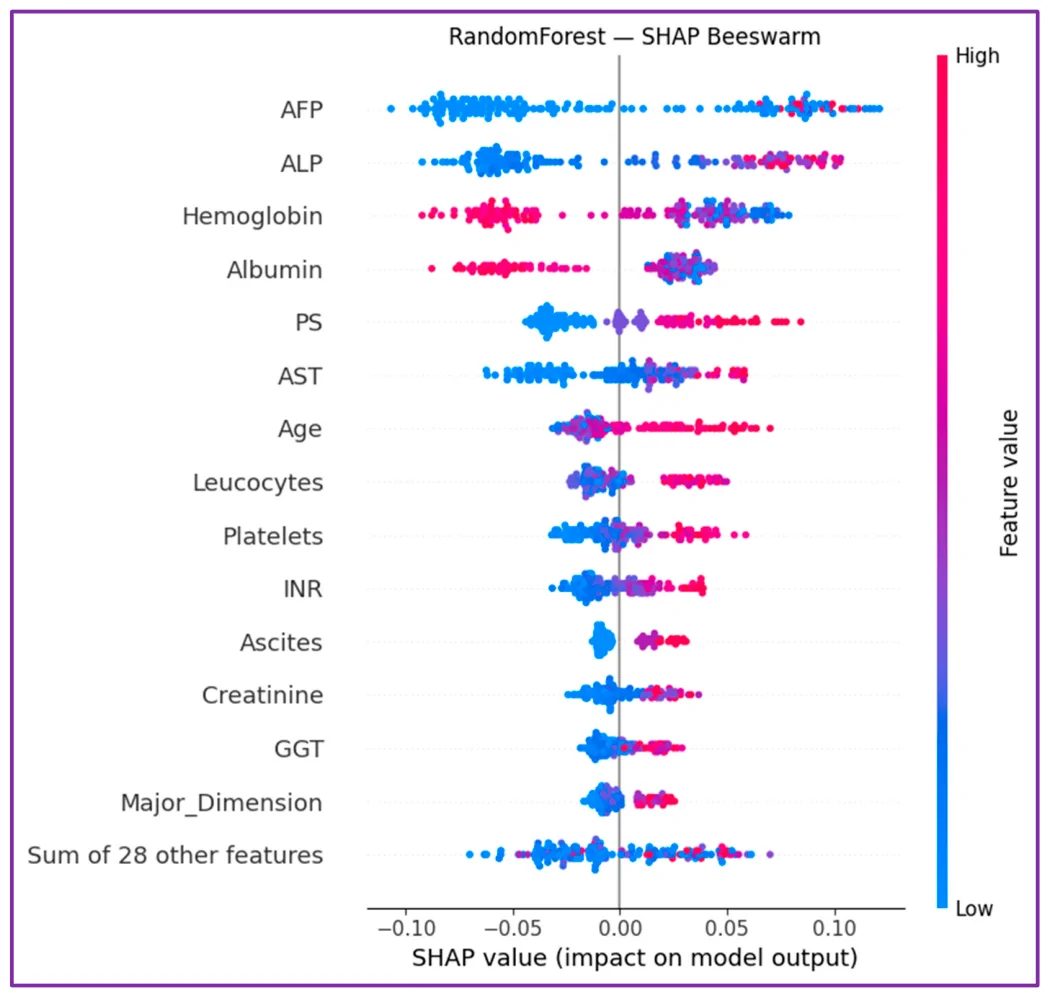
SHAP beeswarm plot of the selected RF model. The model included 42 predictors. Fourteen predictors are displayed individually, while the remaining 28 predictors are aggregated as “sum of 28 other predictors.” In this figure, Major_Dimension denotes the maximum diameter of the largest nodule, measured in centimeters.

**Figure 6:**
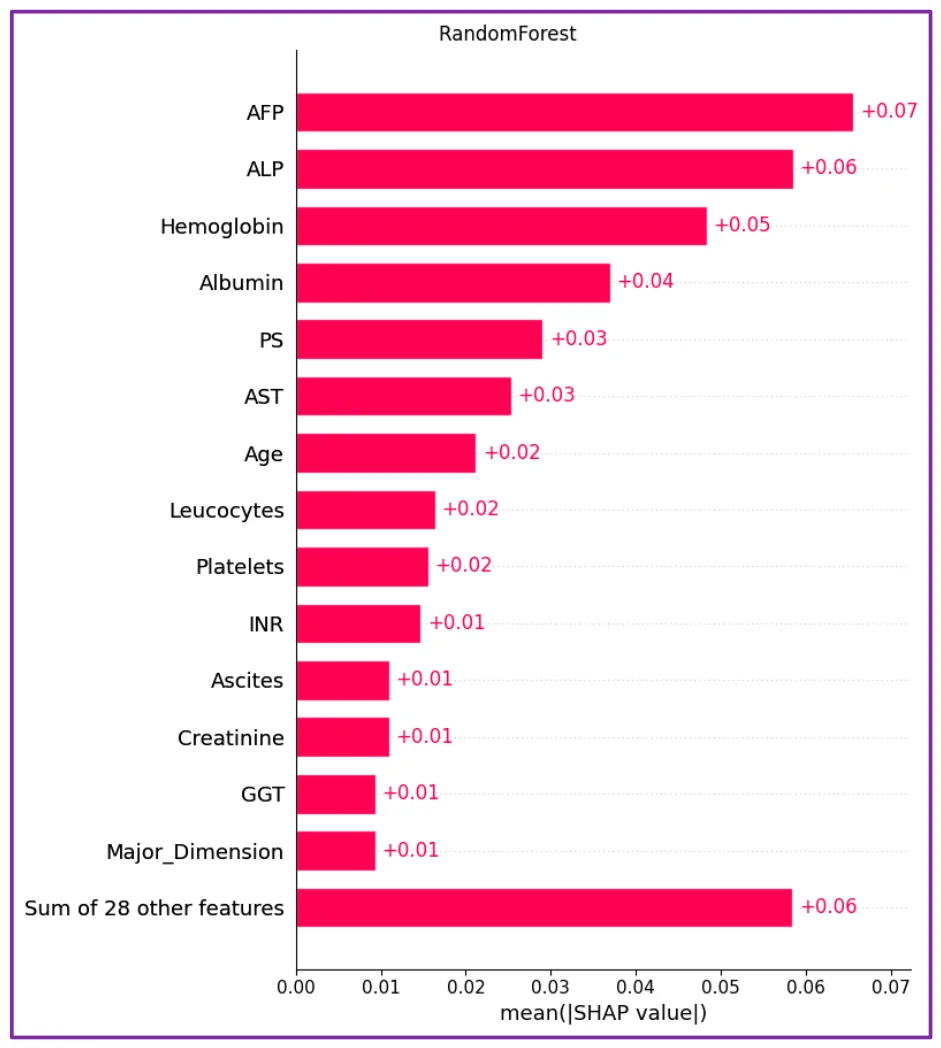
Global predictor importance in the selected RF model based on mean absolute SHAP values. The model included 42 predictors. Fourteen predictors are displayed individually, while the remaining 28 predictors are aggregated as “sum of 28 other predictors.” In this figure, Major_Dimension denotes the maximum diameter of the largest nodule, measured in centimeters.

SHAP was used to interpret the final RF model, which was selected based on nCV performance and subsequently refitted using all 165 patients. Global feature importance, quantified by the mean absolute SHAP value, identified AFP as the strongest individual contributor to model predictions, followed by ALP, hemoglobin, albumin, performance status, and AST. Additional influential predictors included age, leukocyte count, platelet count, INR, ascites, creatinine, GGT, and maximum diameter of the largest nodule (Figure 5 and Figure 6). The SHAP beeswarm plot further illustrated the direction of individual predictor contributions. Higher AFP and ALP values generally shifted predictions toward a higher probability of one-year mortality, whereas higher hemoglobin and albumin values generally shifted predictions toward a lower probability of mortality. Higher performance-status scores, AST, age, leukocyte count, platelet count, INR, ascites severity, and several other clinical variables also tended to shift model predictions toward higher mortality probability. Overall, the SHAP analysis indicated that the final RF model relied on a combination of tumor-related, hepatic, hematologic, and general clinical characteristics. SHAP values describe contributions to the fitted model’s predictions and should not be interpreted as evidence of independent prognostic effects or causal relationships. This analysis therefore provides insight into model behavior rather than clinical causation.

To further assess the robustness of feature importance, SHAP stability was evaluated across the five outer folds of nCV (Supplementary Table S2). AFP, ALP, hemoglobin, and albumin were consistently ranked among the top five predictors in all five outer folds, with mean ranks of 1.8 ± 1.3, 2.0 ± 0.7, 3.2 ± 0.8, and 3.8 ± 1.8, respectively. Performance status was among the top 10 predictors in all five folds and among the top five in three folds, whereas AST was among the top 10 predictors in four of five folds. These findings indicate that the highest-ranked predictors showed relatively consistent importance across different training subsets, although greater rank variability was observed for some lower-ranked features.

### 3.7. Comparison with Penalized Logistic Regression

To provide a conventional statistical benchmark, an L2-penalized logistic regression (LR) model was additionally evaluated using the same nested cross-validation framework with five outer folds and five inner folds. The same predictors used for the tree-based models were included in the LR model, and all preprocessing steps were restricted to the corresponding training partitions. Class imbalance was addressed using balanced class weights, and the regularization strength was optimized within the inner cross-validation loop. Penalized LR achieved a mean outer-fold ROC-AUC of 0.775 ± 0.090, a mean PR-AUC of 0.673 ± 0.107, and a mean Brier score of 0.224 ± 0.025. Based on the pooled out-of-fold predictions from all 165 patients, the ROC-AUC was 0.772 and the PR-AUC was 0.656 (Table 8).

Overall, penalized LR showed discrimination comparable to that of the three tree-based models. The mean ROC-AUC ranged from 0.773 to 0.788 across all four models, with RF yielding the highest value (0.788) and penalized LR achieving 0.775. Similarly, mean PR-AUC values were closely comparable, ranging from 0.669 to 0.673. Penalized LR also achieved the highest pooled out-of-fold PR-AUC (0.656), although the differences were small. In contrast, its mean Brier score (0.224) was higher than those of RF (0.188), XGBoost (0.194), and CatBoost (0.203), indicating greater mean squared prediction error in this dataset. Collectively, these findings do not demonstrate a clear discrimination advantage of the tree-based ML models over penalized LR in this dataset, although the tree-based models showed lower Brier scores.

## 4. Discussion

In the present study, we developed and internally validated three ML models for predicting one-year mortality in patients with HCC and examined how estimated model performance varied according to the internal-validation strategy. The principal finding was that both the magnitude of estimated performance and, to some extent, the relative ranking of the algorithms differed between the two validation frameworks. In the conventional 80/20 train–test analysis, RF and CatBoost achieved the highest ROC-AUC, whereas under the prespecified primary nCV framework, RF achieved the highest mean outer-fold ROC-AUC and was therefore selected as the final model. Nevertheless, the differences among RF, CatBoost, and XGBoost under nCV were small, and the present data do not support a strong claim of algorithmic superiority. Rather, these findings indicate that, in a modest-sized clinical dataset, interpretation of model performance should account not only for the algorithm used but also for the validation framework from which the performance estimate and its associated uncertainty are derived.

The comparison between the two validation strategies provides an important methodological perspective on the observed model performance. In the conventional 80/20 split, performance was evaluated in a single holdout sample comprising only 33 patients, and the resulting ROC-AUC estimates were higher than those obtained from pooled out-of-fold predictions under nCV. The relative ranking of the three algorithms also differed between the two validation frameworks. This sensitivity to data partitioning is consistent with the findings of An et al. [18], who demonstrated substantial variation in estimated ML performance across repeated random train–test partitions, particularly in small-sample settings [18]. More broadly, validation procedures can differ in their susceptibility to overfitting and optimistic performance estimation. Vabalas et al. [20] showed that the choice and implementation of validation strategy can materially affect estimated classification performance, particularly when model-development procedures are not adequately separated from model evaluation [20]. nCV provides such separation by restricting model-development steps to the inner resampling procedure and reserving the outer folds for performance assessment; this structure is particularly relevant when data-dependent procedures such as model selection, feature selection, or hyperparameter optimization are performed [19]. These considerations are consistent with broader guidance for clinical prediction modeling, which emphasizes the risks of overfitting and unreliable performance estimation when the available sample is limited relative to the modeling and evaluation process and recommends rigorous internal validation of predictive performance [15,16,25]. More recent oncology-specific guidance similarly identifies small sample size as a source of model instability and unreliable performance estimates and recommends resampling-based approaches such as bootstrapping or cross-validation for internal validation [26]. In the present study, bootstrap confidence intervals were narrower for the pooled nCV estimates than for the single-split estimates. This difference should be interpreted in the context of the unequal evaluation sample sizes, as the pooled nCV analysis incorporated out-of-fold predictions from all 165 patients, whereas the conventional analysis was based on a 33-patient test set. Therefore, the observed difference in interval widths likely reflects, at least partly, the larger number of patients contributing to the pooled nCV estimates rather than an inherent gain in precision attributable to the validation strategy itself. Accordingly, the confidence-interval widths are interpreted as descriptive measures of uncertainty within each validation framework.

An important context for interpreting the present findings is provided by the previous analysis of the same HCC Survival dataset from the UCI Machine Learning Repository by Chicco and Oneto [27]. Using the same cohort of 165 patients, they identified ALP, AFP, and hemoglobin among the most predictive features for one-year survival using Random-Forest-based feature-ranking procedures [27]. The present analysis showed notable concordance with these findings, as AFP, ALP, and hemoglobin were again among the most influential features in the final RF model, despite the use of a different model-interpretation framework based on SHAP. Because both studies were derived from the same underlying patient cohort, however, this concordance should not be interpreted as independent replication or external confirmation of these prognostic signals. Rather, the recurrence of these variables across different modeling and feature-importance approaches suggests that their predictive contribution is relatively persistent within this particular dataset. This distinction is important because feature importance reflects the contribution of a variable to predictions generated by the fitted model and does not establish an independent prognostic effect or a causal relationship. The present analysis therefore extends the earlier work primarily by addressing a different methodological question: rather than focusing principally on survival classification and feature ranking, we directly compared a conventional single train–test split with nCV while holding the surrounding modeling pipeline as constant as possible, evaluated three tree-based ensemble algorithms, generated pooled out-of-fold predictions, and assessed discrimination, calibration, clinical utility, and uncertainty within the same analytical framework.

The clinical profile of the features highlighted by the final RF model was also broadly consistent with established prognostic domains in HCC. AFP, one of the most influential features in our SHAP analysis, is an established tumor marker in HCC and has been associated with adverse survival outcomes; a meta-analysis by Chen et al. [28], for example, demonstrated an association between elevated serum AFP and poorer overall and recurrence-free survival following surgical resection [28]. Liver-related variables were also prominent in our model. In particular, albumin and ALP may reflect complementary liver-related information, and their combined prognostic relevance is supported by studies of the albumin-to-alkaline phosphatase ratio (AAPR). A recent meta-analysis found higher AAPR values to be associated with more favorable overall survival in patients with HCC, including in the pooled multivariable analysis (hazard ratio [HR], 0.476) [29]. Consistent with this evidence, Yousif and Bakosh [30] reported that AAPR showed discriminatory ability for one-year mortality in a retrospective cohort of 188 patients with cirrhotic HCC (AUC 0.704), although AAPR did not retain statistical significance in their multivariable Cox model. Performance status represents another clinically distinct prognostic domain. In a large HCC cohort, Hsu et al. [31] demonstrated progressively poorer long-term survival with worsening Eastern Cooperative Oncology Group performance status and identified performance status as an independent prognostic predictor [31]. Importantly, the simultaneous prominence of tumor-related, liver-related, and patient-related variables in our model is conceptually consistent with established multidimensional HCC prognostic frameworks. For example, the externally validated ITA.LI.CA prognostic system integrates tumor stage, Child–Pugh score, ECOG performance status, and AFP to estimate individual survival [32]. Thus, rather than suggesting that any single SHAP-ranked variable independently determines outcome, our findings indicate that the model derived its predictions from information spanning several clinically relevant prognostic domains. This clinical concordance supports the plausibility of the model’s internal prediction structure but should not be interpreted as evidence of independent prognostic effects or causal relationships.

The present findings should also be considered within the increasingly diverse literature on ML-based prognostic modeling in HCC. Contemporary studies have addressed substantially different clinical populations and prediction settings, including multicenter cohorts with external validation [4], AFP-positive HCC derived from population-based registry data [11], advanced multiple HCC [12], small postoperative cohorts [6], patients spanning different disease stages [7], large HCC following curative hepatectomy [5], and survival modeling based on large registry and genomic datasets [14]. These studies illustrate the breadth of contemporary ML-based HCC prognostication, but their reported performance estimates are not directly comparable because the target populations, outcome definitions, prediction horizons, candidate predictors, model classes, and validation procedures differ substantially. The algorithms identified as best-performing also vary across these settings, including Random Survival Forest, XGBoost, gradient-boosting methods, support vector machines, and ensemble survival approaches, further emphasizing the context dependence of model performance. This context dependence is also evident in dynamic prognostication; using longitudinal time-series data from 2511 patients with HCC, Shen et al. [13] showed that relative predictive performance varied according to the prediction and evaluation time points. More recently, Lim et al. [33] developed and validated an ML prognostic model in 1102 patients with HCC from five international tertiary centers, using separate derivation, internal-validation, and external-validation cohorts and evaluating both discrimination and calibration. Jin et al. [34] similarly reported a multicenter prognostic modeling study in patients with HCC treated with transarterial chemoembolization, incorporating external validation in an independent cohort. Consequently, differences in reported ROC-AUC, C-index, or other performance measures across studies cannot be attributed solely to the underlying learning algorithm.

Our findings provide a controlled illustration of this broader methodological issue: even when the patient cohort, predictors, algorithms, preprocessing procedures, and optimization strategy were held constant, changing the internal-validation framework altered the magnitude of estimated performance and, to some extent, the relative ranking of the models. Accordingly, comparisons of ML algorithms across HCC studies should consider the validation design and overall analytical framework alongside the reported discrimination metrics rather than interpreting numerical differences as direct evidence of algorithmic superiority. From this perspective, the broadly similar performance of RF, XGBoost, and CatBoost under nCV in the present study may be more informative than the identification of a single numerically highest-performing algorithm.

Beyond discrimination, the present study also evaluated calibration and decision-analytic performance, because satisfactory discrimination alone does not establish that predicted probabilities are well calibrated or that using a model to guide decisions would provide clinical benefit. Contemporary methodological guidance recommends evaluating these complementary dimensions separately, with calibration assessed across the range of predicted risks using graphical calibration methods [35,36]. Recent oncology-specific guidance similarly emphasizes that prediction-model evaluation should extend beyond discrimination to include calibration and assessment of clinical utility [26]. In our analysis, the pooled out-of-fold calibration results showed model-dependent deviations from ideal calibration, particularly across intermediate and higher predicted probabilities. Decision curve analysis provided an additional perspective by comparing the net benefit of model-guided classification with default treat-all and treat-none strategies across the evaluated range of threshold probabilities. This distinction is important because a model may show good discrimination and calibration yet still provide limited value for clinical decision-making; decision-analytic methods explicitly incorporate the consequences of alternative decisions through threshold-dependent net benefit [25,37]. In the present study, all three models showed greater net benefit than the default strategies over portions of the evaluated threshold range, although the magnitude and range of this advantage varied among the models. Multidimensional evaluation incorporating discrimination, calibration, and decision-curve analysis has also been adopted in recent HCC ML research; for example, Nie et al. [38] evaluated these domains jointly when comparing Random Survival Forest, DeepSurv, and Cox proportional-hazards models in patients with HCC and second primary malignancies. Nevertheless, our decision-curve findings should be interpreted as exploratory and should not be considered evidence of established clinical utility, clinical effectiveness, or readiness for treatment guidance. Clinically actionable threshold probabilities were not prospectively specified, and neither calibration nor net benefit has yet been evaluated in an independent external population.

Although RF was selected as the final model according to the prespecified primary criterion of mean outer-fold ROC-AUC, this selection should be distinguished from evidence of algorithmic superiority. The differences among the three models under nCV were small: mean outer-fold ROC-AUC was 0.788 for RF, 0.784 for CatBoost, and 0.773 for XGBoost, while XGBoost achieved a marginally higher mean PR-AUC than RF. Moreover, no formal pairwise hypothesis testing was performed to establish whether the observed differences between algorithms exceeded that expected from sampling variability. RF should therefore be regarded as the model selected by the prespecified decision rule for subsequent interpretation, rather than as a demonstrably superior algorithm for HCC prognostication. The context-specific nature of model selection is also illustrated by contemporary HCC studies; for example, Nie et al. [38] reported that Random Survival Forest outperformed Cox proportional-hazards and DeepSurv models in a cohort of 1580 patients with HCC and second primary malignancies, although their population, time-to-event outcome structure, predictors, and modeling framework differed substantially from those of the present study [38]. Importantly, Random Survival Forest is a survival-analysis method and should not be considered equivalent to the RF classifier evaluated here. Accordingly, the selection of RF in our analysis should be interpreted as a dataset-, outcome-, and validation-specific modeling decision. Its principal role was to identify, according to a prespecified criterion, the model to be carried forward for interpretation and potential future external validation.

### 4.1. Strengths

Several methodological features strengthen the interpretation of this study. First, the three ML algorithms were evaluated within a common analytical pipeline, allowing performance to be compared while keeping preprocessing, class-imbalance handling, hyperparameter optimization, threshold selection, and performance assessment consistent across algorithms. The study also directly compared two internal-validation strategies under otherwise comparable conditions, allowing the influence of validation design on estimated performance and uncertainty to be examined more directly than through comparisons across independently developed models.

Second, nCV was prespecified as the primary internal-validation framework, with hyperparameter optimization and threshold selection confined to training procedures and performance evaluated in held-out outer folds. Preprocessing was likewise restricted to training partitions. This separation between model development and performance assessment reduced opportunities for information leakage and optimization-induced optimism and ensured that primary performance estimates were based on predictions from observations not used in model development.

Third, model performance was evaluated multidimensionally rather than relying exclusively on ROC-AUC. Discrimination was complemented by PR-AUC, threshold-dependent classification metrics, Brier scores, calibration curves, and decision curve analysis, providing information on ranking ability, probabilistic accuracy, classification performance, and potential clinical net benefit. Bootstrap confidence intervals were also reported for principal performance measures, allowing uncertainty to be considered alongside point estimates. Confidence-interval widths were compared descriptively between validation frameworks; however, because these intervals were derived from test-set or pooled out-of-fold predictions rather than repetition of the complete model-development process, they should not be interpreted as evidence of an inherent precision advantage of either validation strategy.

Finally, SHAP was used to characterize how individual features contributed to predictions from the selected RF model. SHAP values were interpreted as contributions to model predictions rather than estimates of independent prognostic effects or causal relationships, allowing examination of the model’s internal prediction structure without causal or etiological interpretation. The use of a publicly available dataset, unified modeling pipelines, and a fixed random seed further supports reproducibility and facilitates independent methodological scrutiny.

### 4.2. Limitations

Several limitations should be considered. First, the study included only 165 patients and a substantial number of candidate predictors relative to the sample size. Although nCV separated model optimization from performance evaluation and preprocessing was confined to training partitions, these measures cannot eliminate uncertainty and model instability associated with limited sample size. Small samples may increase overfitting and produce imprecise or unstable performance estimates. Accordingly, the small differences among RF, XGBoost, and CatBoost should not be interpreted as definitive performance differences, but as estimates subject to sampling variability.

Second, this was a secondary analysis of a retrospective, publicly available dataset not originally collected for the present prediction-modeling objectives. The available predictors, outcome definition, measurement procedures, and missingness patterns were therefore determined by the original data collection, and potentially relevant unmeasured predictors could not be evaluated. Missing values were handled using fold-specific imputation, avoiding use of information from corresponding validation observations when estimating imputation parameters. However, imputation cannot reconstruct unobserved information, and the underlying missing-data mechanism could not be established. Thus, fold-specific imputation limits information leakage but does not remove potential bias or uncertainty from informative or extensive missingness.

Third, the study provides internal rather than external validation. nCV estimates predictive performance within the available cohort but does not establish transportability across institutions, geographic regions, clinical settings, or treatment eras. Contemporary guidance emphasizes that internal validation alone may mask poor generalizability [26]. Therefore, the present estimates should not be interpreted as evidence of clinical effectiveness, transportability, or implementation readiness. Independent external validation in clinically representative HCC populations is required before broader application. Such validation should be adequately sized to estimate discrimination and calibration with sufficient precision and, where meaningful thresholds can be defined, to evaluate clinical utility [35,36].

Fourth, the bootstrap confidence intervals require specific interpretation. They were derived by resampling the available test-set or pooled out-of-fold predictions rather than repeating the complete model-development pipeline, including preprocessing, hyperparameter optimization, threshold selection, and model fitting, within each bootstrap resample. Consequently, these intervals quantify uncertainty around the available performance estimates but do not capture the additional variability arising from the complete modeling procedure. A fully nested resampling approach that repeats the entire model-development pipeline would provide a more comprehensive assessment of total modeling uncertainty, although at substantially greater computational cost. Accordingly, the reported confidence intervals should be interpreted as descriptive measures of uncertainty rather than as estimates of total modeling uncertainty or strong evidence of model stability. Moreover, the narrower intervals observed for the pooled out-of-fold estimates should not be interpreted as evidence of an inherent precision advantage of nCV, because the two validation frameworks were based on different effective evaluation sample sizes.

Fifth, missing data and the limited hyperparameter search should be considered when interpreting the results. Although seven predictors were excluded before model development, 6.32% of values remained missing among the 42 retained predictors, and only 31 patients (18.8%) had complete information across these variables. Missingness was unevenly distributed across predictors. Median imputation for continuous variables and most-frequent-value imputation for binary and ordinal variables were fitted exclusively within the corresponding training partitions to limit information leakage. Nevertheless, these single-imputation methods do not account for uncertainty in the imputed values and may introduce bias if missingness is informative. The underlying missing-data mechanism could not be established, and neither alternative imputation strategies nor missingness indicators were evaluated in sensitivity analyses. Consequently, the extent to which the choice of imputation method affected model performance remains uncertain. Future studies with larger independent cohorts should examine missing-data mechanisms and compare alternative approaches, including multiple or iterative imputation and missingness indicators. In addition, the hyperparameter search was limited to 15 Optuna trials per optimization process. This limit balanced exploration of the predefined search spaces against the computational demands of nested cross-validation, but it may have left potentially better model configurations unexplored. More extensive optimization could be evaluated in future studies with greater computational resources.

Finally, calibration, decision-curve, and SHAP analyses remain exploratory. Pooled out-of-fold calibration curves provided an internal assessment of agreement between predicted and observed mortality probabilities, but calibration has not been independently evaluated. Decision curve analysis showed greater net benefit than default strategies over parts of the threshold range, yet clinically actionable thresholds were not prospectively defined or externally tested; thus, these findings remain exploratory and do not establish clinical utility or clinical effectiveness. SHAP values describe predictor contributions to predictions from the fitted RF model and do not establish independent prognostic effects, biological mechanisms, or causal relationships. Independent confirmation is required before these findings can support clinical use or stronger prognostic inference.

## 5. Conclusions

In this secondary analysis of 165 patients with HCC, estimates of one-year mortality prediction performance differed between a conventional 80/20 train–test split and nCV. Performance also varied across six stratified train–test partitions, illustrating the sensitivity of single-split estimates to patient allocation. Under the prespecified primary nCV framework, RF achieved the highest mean outer-fold ROC-AUC and was selected for model interpretation. However, differences among RF, XGBoost, and CatBoost were small, and the tree-based models showed no clear discrimination advantage over penalized logistic regression. Calibration differed across models, while decision curve analysis showed exploratory net benefit over default strategies across portions of the evaluated threshold range; neither finding establishes clinical utility. SHAP analysis showed that the selected RF model used information from tumor-related, liver-related, hematologic, and functional-status predictors. These contributions describe model predictions and do not establish independent prognostic or causal effects. The comparison between validation frameworks is descriptive because their performance estimates were obtained from evaluation samples of different sizes. Independent external validation in adequately sized and clinically representative HCC cohorts, including reassessment of discrimination, calibration, and decision-analytic performance at clinically defined thresholds, is required before clinical application.:

## Figures and Tables

**Table 1:** Continuous clinical and laboratory characteristics according to one-year survival status.

Variables	Survived at 1 Year	Died Within 1 Year	*p*-Value
Age at diagnosis, years	64.0 [56.0–73.0]	69.0 [60.0–78.0]	0.036
INR	1.3 [1.2–1.5]	1.4 [1.2–1.7]	0.033
AFP (ng/mL)	9.4 [4.9–77.0]	205.5 [26.5–3718.0]	<0.001
Hemoglobin (g/dL)	13.7 [12.0–14.9]	12.2 [10.8–13.3]	<0.001
MCV (fL)	95 [90–100]	94 [89–102]	0.492
Leukocyte count (G/L)	5.8 [4.9–7.6]	6.9 [4.8–9.6]	0.119
Platelet count (G/L)	124 [78–184]	143 [99–261]	0.045
Total protein (g/dL)	7.2 [6.4–7.6]	7.0 [6.3–7.3]	0.151
Albumin (g/dL)	3.7 [3.1–4.2]	3.2 [2.9–3.6]	<0.001
Total bilirubin (mg/dL)	1.3 [0.8–2.4]	1.7 [1.0–3.8]	0.033
Direct bilirubin (mg/dL)	0.6 [0.3–1.0]	1.2 [0.6–2.3]	0.001
ALT (U/L)	46 [31–74]	53 [28–82]	0.663
AST (U/L)	61 [40–94]	85 [58–127]	0.002
GGT (U/L)	163 [82–277]	236 [115–464]	0.019
ALP (U/L)	129 [97–182]	222 [147–350]	<0.001
Creatinine (mg/dL)	0.8 [0.7–1.0]	0.9 [0.7–1.3]	0.097
Number of hepatic nodules (0–5)	2.0 [1.0–5.0]	2.0 [1.0–5.0]	0.227
Maximum diameter of the largest nodule (cm)	4.7 [2.6–7.5]	7.1 [3.2–10.0]	0.028
Serum iron (mcg/dL)	92 [55–144]	53 [37–105]	0.009
Ferritin (ng/mL)	239 [77–363]	538 [175–844]	0.002
Alcohol intake (grams/day)	100 [80–100]	100 [75.0–100]	0.483
Cumulative cigarette exposure (packs/year)	35.8 [19.0–48.0]	30.0 [20.0–60.0]	0.291

**Table 2:** Categorical clinical characteristics according to one-year survival status.

Variables	Survived at 1 Year	Died Within 1 Year	*p*-Value
Male sex	81 (79.4)	52 (82.5)	0.771
Symptoms at presentation	44 (51.8)	50 (80.6)	0.001
Smoking	42 (53.8)	21 (45.7)	0.487
Alcohol consumption	74 (72.5)	48 (76.2)	0.737
HBsAg (+)	11 (11.6)	5 (9.4)	0.899
HBeAg (+)	0 (0.0)	1 (2.2)	0.368
Anti-HBc (+)	26 (28.6)	12 (24.0)	0.699
Anti-HCV (+)	18 (18.2)	16 (28.1)	0.215
Cirrhosis	93 (91.2)	56 (88.9)	0.832
Endemic countries	8 (10.3)	2 (4.2)	0.316
Diabetes mellitus	30 (30.3)	26 (41.3)	0.207
Obesity	13 (13.5)	7 (11.9)	0.956
Hemochromatosis	4 (4.2)	3 (6.4)	0.685
Hypertension	39 (39.0)	20 (32.3)	0.485
Chronic renal insufficiency	10 (10.0)	10 (15.9)	0.386
HIV (+)	2 (2.1)	1 (1.8)	1.000
NASH	6 (6.7)	2 (3.8)	0.710
Esophageal varices	49 (62.8)	20 (57.1)	0.716
Splenomegaly	51 (54.8)	33 (57.9)	0.714
PHT	65 (69.9)	45 (73.8)	0.735
PVT	15 (15.2)	21 (33.3)	0.012
Radiologic hallmark of HCC	69 (69.0)	42 (66.7)	0.890
Performance status (active)	63 (61.8)	17 (27.0)	<0.001
Performance status (restricted)	16 (15.7)	14 (22.2)	
Performance status (ambulatory)	17 (16.7)	15 (23.8)	
Performance status (self-care)	6 (5.9)	12 (19.0)	
Performance status (disabled)	0 (0.0)	5 (7.9)	
Hepatic encephalopathy (none)	90 (89.1)	52 (82.5)	0.049
Hepatic encephalopathy (grade I–II)	11 (10.9)	7 (11.1)	
Hepatic encephalopathy (grade III–IV)	0 (0.0)	4 (6.3)	
Ascites degree (none)	76 (76.0)	33 (52.4)	0.004
Ascites degree (mild)	18 (18.0)	18 (28.6)	
Ascites degree (moderate–severe)	6 (6.0)	12 (19.0)	

HBsAg, hepatitis B surface antigen; HBeAg, hepatitis B e antigen; HBcAb, hepatitis B core antibody; HCV, hepatitis C virus; HIV, human immunodeficiency virus; NASH, nonalcoholic steatohepatitis. Percentages were calculated using available non-missing observations for each variable within each outcome group. Therefore, denominators vary across variables. Missing observations were excluded from percentage calculations and statistical comparisons.

**Table 3:** Test-set performance from the single split.

Metric	RF	XGBoost	CatBoost
AUC	0.892	0.862	0.892
PR-AUC	0.860	0.755	0.791
Balanced Accuracy	0.825	0.875	0.875
F1 Score	0.788	0.839	0.839
Accuracy	0.788	0.849	0.849
MCC	0.650	0.736	0.736
Sensitivity	1.000	1.000	1.000
Specificity	0.650	0.750	0.750
PPV	0.650	0.722	0.722
NPV	1.000	1.000	1.000
Brier Score	0.144	0.131	0.159

AUC, area under the receiver operating characteristic curve; PR-AUC, area under the precision–recall curve; MCC, Matthews correlation coefficient; PPV, positive predictive value; NPV, negative predictive value; RF, Random forest.

**Table 4:** Performance variability across different stratified 80/20 train–test partitions.

Model	Seed	ROC-AUC	PR-AUC	Brier Score	Balanced Accuracy	MCC
RF	42	0.892	0.860	0.144	0.825	0.650
	7	0.777	0.718	0.193	0.662	0.354
	19	0.788	0.631	0.198	0.698	0.396
	63	0.731	0.741	0.199	0.648	0.307
	88	0.827	0.724	0.168	0.644	0.293
	123	0.735	0.697	0.216	0.685	0.362
	Mean ± SD	0.792 ± 0.061	0.729 ± 0.075	0.187 ± 0.026	0.694 ± 0.068	0.394 ± 0.131
	Min–Max	0.731–0.892	0.631–0.860	0.144–0.216	0.644–0.825	0.293–0.650
XGBoost	42	0.862	0.755	0.131	0.875	0.736
	7	0.804	0.748	0.190	0.662	0.354
	19	0.658	0.612	0.299	0.585	0.175
	63	0.746	0.751	0.192	0.644	0.293
	88	0.827	0.749	0.170	0.683	0.365
	123	0.719	0.591	0.228	0.698	0.396
	Mean ± SD	0.769 ± 0.076	0.701 ± 0.077	0.202 ± 0.057	0.691 ± 0.098	0.386 ± 0.188
	Min–Max	0.658–0.862	0.591–0.755	0.131–0.299	0.585–0.875	0.175–0.736
CatBoost	42	0.892	0.791	0.159	0.875	0.736
	7	0.765	0.691	0.186	0.748	0.487
	19	0.738	0.649	0.247	0.660	0.316
	63	0.673	0.599	0.287	0.596	0.190
	88	0.819	0.713	0.166	0.735	0.459
	123	0.677	0.590	0.318	0.621	0.238
	Mean ± SD	0.761 ± 0.085	0.672 ± 0.076	0.227 ± 0.067	0.706 ± 0.103	0.404 ± 0.201
	Min–Max	0.673–0.892	0.590–0.791	0.159–0.318	0.596–0.875	0.190–0.736

AUC, area under the receiver operating characteristic curve; PR-AUC, area under the precision–recall curve; MCC, Matthew’s correlation coefficient; PPV, positive predictive value; Min-Max, minimum-maximum; NPV, negative predictive value; RF, Random Forest; SD, standard deviation.

**Table 5:** Mean outer-fold performance from nCV with five outer folds and five inner folds (mean ± SD).

Metric	RF	XGBoost	CatBoost
AUC	0.788 ± 0.064	0.773 ± 0.042	0.784 ± 0.085
PR-AUC	0.671 ± 0.070	0.673 ± 0.018	0.669 ± 0.121
Balanced Accuracy	0.698 ± 0.076	0.686 ± 0.039	0.694 ± 0.104
F1 Score	0.644 ± 0.072	0.629 ± 0.046	0.638 ± 0.124
Accuracy	0.691 ± 0.108	0.679 ± 0.063	0.685 ± 0.113
MCC	0.397 ± 0.149	0.375 ± 0.082	0.385 ± 0.202
Sensitivity	0.715 ± 0.086	0.714 ± 0.121	0.728 ± 0.161
Specificity	0.680 ± 0.205	0.658 ± 0.163	0.659 ± 0.172
PPV	0.607 ± 0.131	0.582 ± 0.102	0.581 ± 0.133
NPV	0.792 ± 0.029	0.796 ± 0.037	0.803 ± 0.079
Brier Score	0.188 ± 0.027	0.194 ± 0.035	0.203 ± 0.053

AUC, area under the receiver operating characteristic curve; PR-AUC, area under the precision–recall curve; MCC, Matthew’s correlation coefficient; PPV, positive predictive value; NPV, negative predictive value; SD, standard deviation.

**Table 6:** Comparison of bootstrap 95% confidence intervals and interval widths for the primary metrics.

Model	Metric	nCV (Pooled Out-of-Fold Predictions)	Width	Conventional Split (Test-Set Predictions)	Width
RF	AUC	0.784 (0.711–0.851)	0.140	0.892 (0.776–0.979)	0.203
	PR-AUC	0.637 (0.521–0.777)	0.256	0.860 (0.687–0.971)	0.284
	Brier	0.188 (0.166–0.211)	0.045	0.144 (0.099–0.191)	0.092
XGBoost	AUC	0.772 (0.699–0.841)	0.142	0.862 (0.722–0.975)	0.253
	PR-AUC	0.646 (0.529–0.781)	0.252	0.755 (0.524–0.966)	0.442
	Brier	0.194 (0.160–0.231)	0.071	0.131 (0.049–0.221)	0.172
CatBoost	AUC	0.775 (0.701–0.840)	0.139	0.892 (0.765–0.988)	0.223
	PR-AUC	0.648 (0.530–0.773)	0.243	0.791 (0.574–0.984)	0.410
	Brier	0.203 (0.163–0.247)	0.084	0.159 (0.068–0.253)	0.185

nCV, nested cross-validation; CI, confidence interval; ROC-AUC, area under the receiver operating characteristic curve; PR-AUC, area under the precision–recall curve.

**Table 7:** Sensitivity analysis comparing weighted and unweighted models under nCV.

Model	RF	RF	XGBoost	XGBoost	CatBoost	CatBoost
Analysis	Weighted	Unweighted	Weighted	Unweighted	Weighted	Unweighted
Mean ROC-AUC ± SD	0.788 ± 0.064	0.808 ± 0.076	0.773 ± 0.042	0.780 ± 0.059	0.784 ± 0.085	0.805 ± 0.088
Mean PR-AUC ± SD	0.671 ± 0.070	0.691 ± 0.096	0.673 ± 0.018	0.698 ± 0.052	0.669 ± 0.121	0.719 ± 0.112
Mean Brier ± SD	0.188 ± 0.027	0.184 ± 0.022	0.194 ± 0.035	0.192 ± 0.028	0.203 ± 0.053	0.190 ± 0.035
Pooled ROC-AUC	0.784	0.793	0.772	0.767	0.775	0.784
Pooled PR-AUC	0.637	0.625	0.646	0.641	0.648	0.672
Pooled Brier	0.188	0.184	0.194	0.192	0.203	0.190
Calibration Intercept	−0.270	0.191	−0.355	−0.056	−0.002	−0.006
Calibration Slope	1.438	1.571	0.691	0.748	0.427	0.574

RF, Random forest; ROC-AUC, area under the receiver operating characteristic curve; PR-AUC, area under the precision–recall curve; SD, standard deviation. Mean values and SDs were calculated across the five outer folds of nCV, whereas pooled metrics and calibration estimates were calculated from pooled out-of-fold predictions.

**Table 8:** Comparison of penalized logistic regression with the tree-based models under nCV.

Metric	Penalized LR	RF	XGBoost	CatBoost
Mean ROC-AUC ± SD	0.775 ± 0.090	0.788 ± 0.064	0.773 ± 0.042	0.784 ± 0.085
Mean PR-AUC ± SD	0.673 ± 0.107	0.671 ± 0.070	0.673 ± 0.018	0.669 ± 0.121
Mean Brier Score ± SD	0.224 ± 0.025	0.188 ± 0.027	0.194 ± 0.035	0.203 ± 0.053
Pooled out-of-fold ROC-AUC	0.772	0.784	0.772	0.775
Pooled out-of-fold PR-AUC	0.656	0.637	0.646	0.648

## Data Availability

The HCC Survival dataset analyzed in this study is publicly available from the UCI Machine Learning Repository at https://doi.org/10.24432/C5TS4S. The dataset was originally provided by Santos et al. and is available under the Creative Commons Attribution 4.0 International (CC BY 4.0) license.

## Supplementary Materials

The following supporting information can be downloaded at: https://www.mdpi.com/article/10.3390/jcm15207899/s1, Table S1: Predictor-specific missingness among the 42 predictors retained for model development; Table S2: Stability of SHAP-based predictor importance across the five outer folds of nCV; Figure S1: ROC curves for the conventional 80/20 test set; Figure S2: Precision–recall curves for the conventional 80/20 test set; Figure S3: Calibration curves for the conventional 80/20 test set; Figure S4: Decision curve analysis for the conventional 80/20 test set; Figure S5: SHAP summary plot of the RF model for the conventional 80/20 test set; Figure S6: Global predictor importance based on mean absolute SHAP values of the RF model for the conventional 80/20 test set.
