# Supplementary material for: Comparison of Internal Validation Strategies for Estimating Machine Learning Model Performance in One-Year Mortality Prediction in Hepatocellular Carcinoma
Source: J Clin Med. 2026 Oct 13;15(20):7899. doi: 10.3390/jcm15207899 (PMC13706229; doi:10.3390/jcm15207899)
Supplement: Supplementary file 1 [file jcm-15-07899-s001.zip › jcm-4561806-supplementary/supplementary figures.pdf]

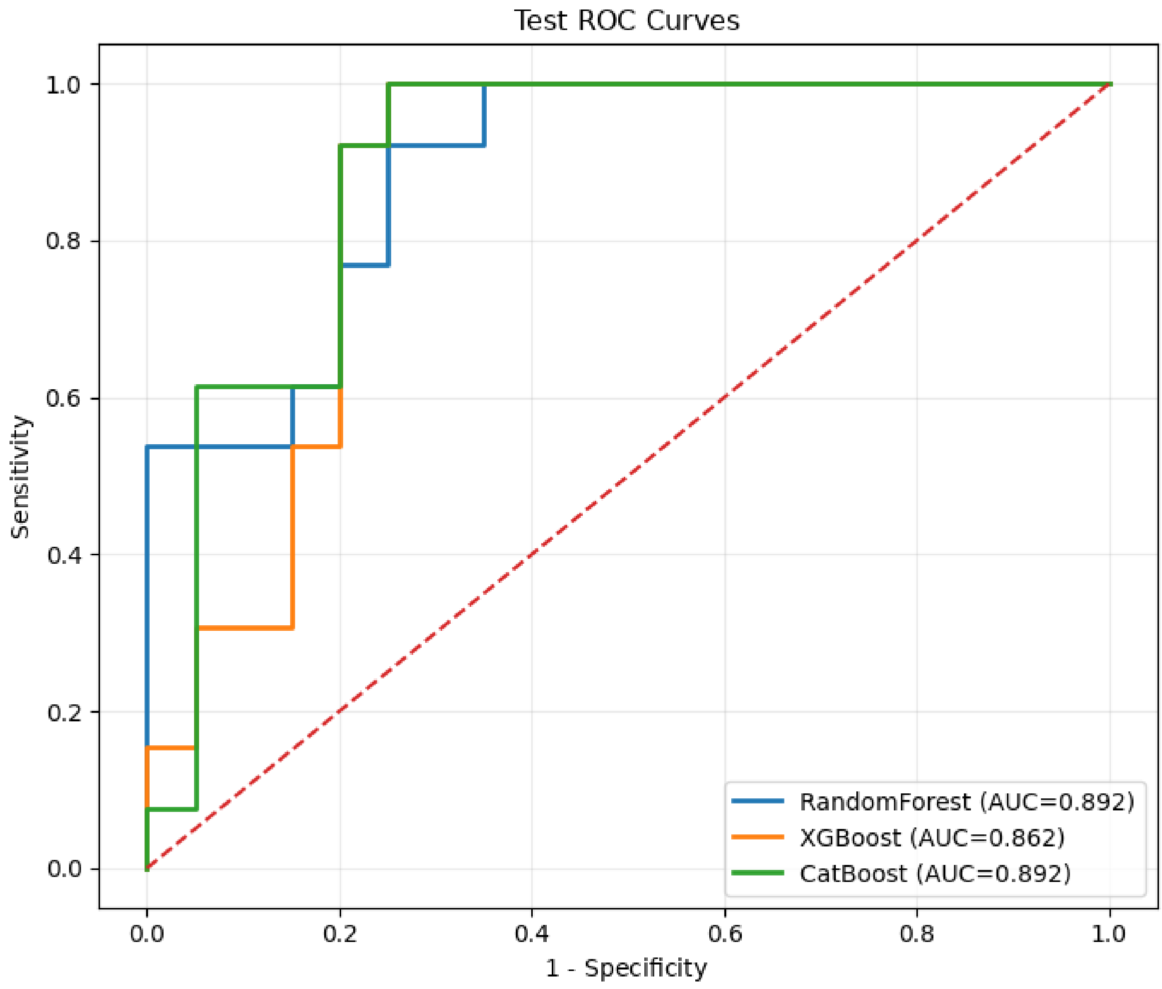

Figure S1. ROC curves for the conventional 80/20 test set.

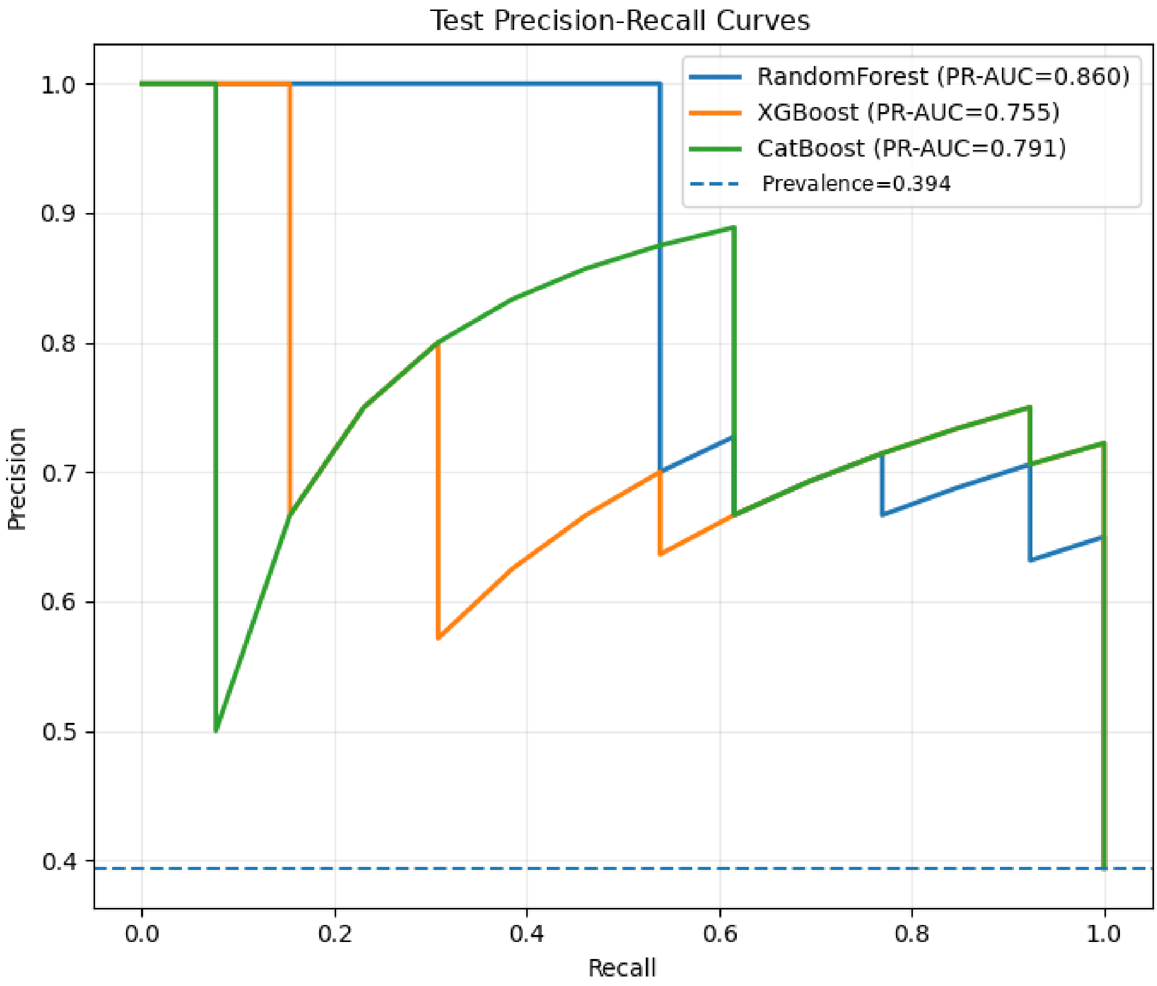

Figure S2. Precision–recall curves for the conventional 80/20 test set.

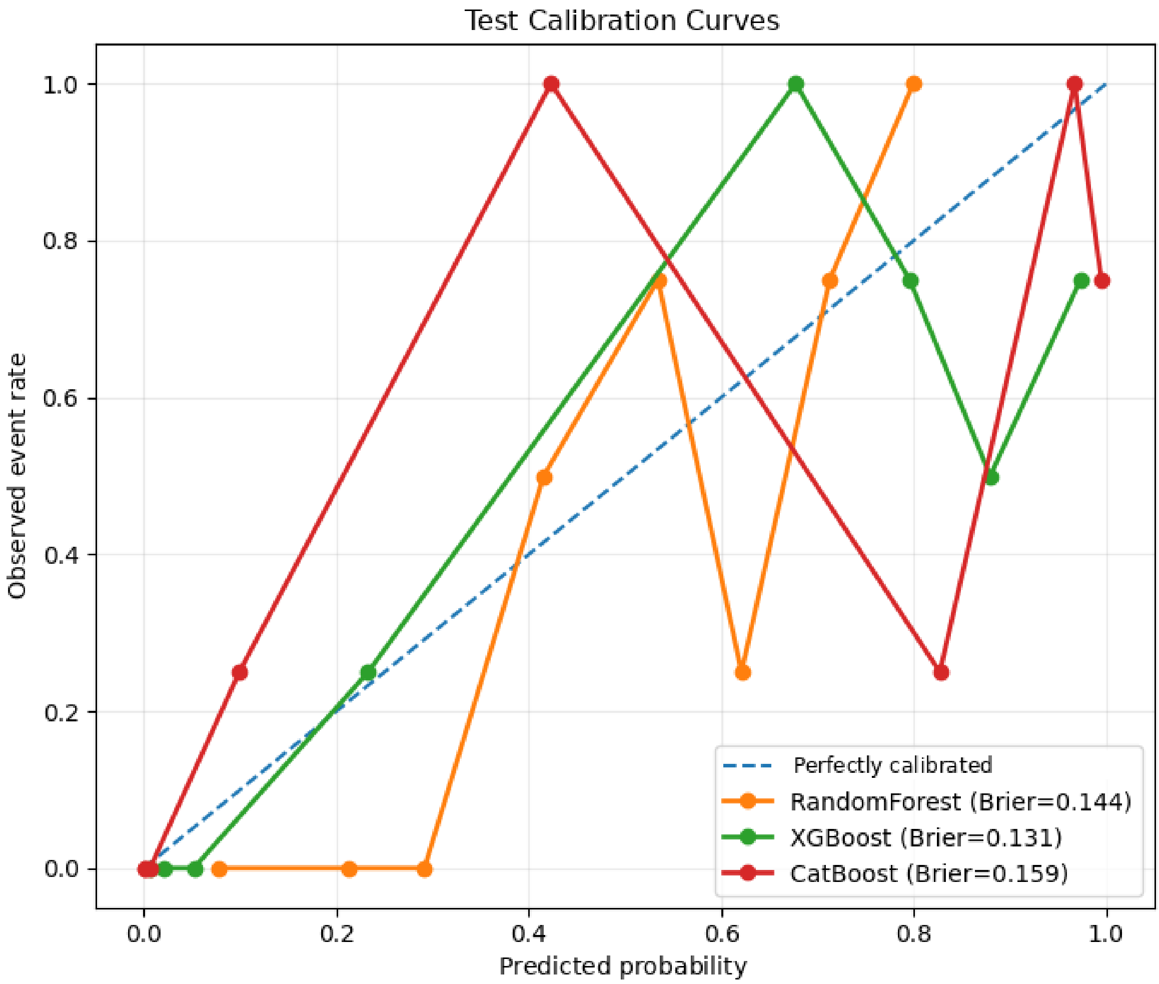

Figure S3. Calibration curves for the conventional 80/20 test set.

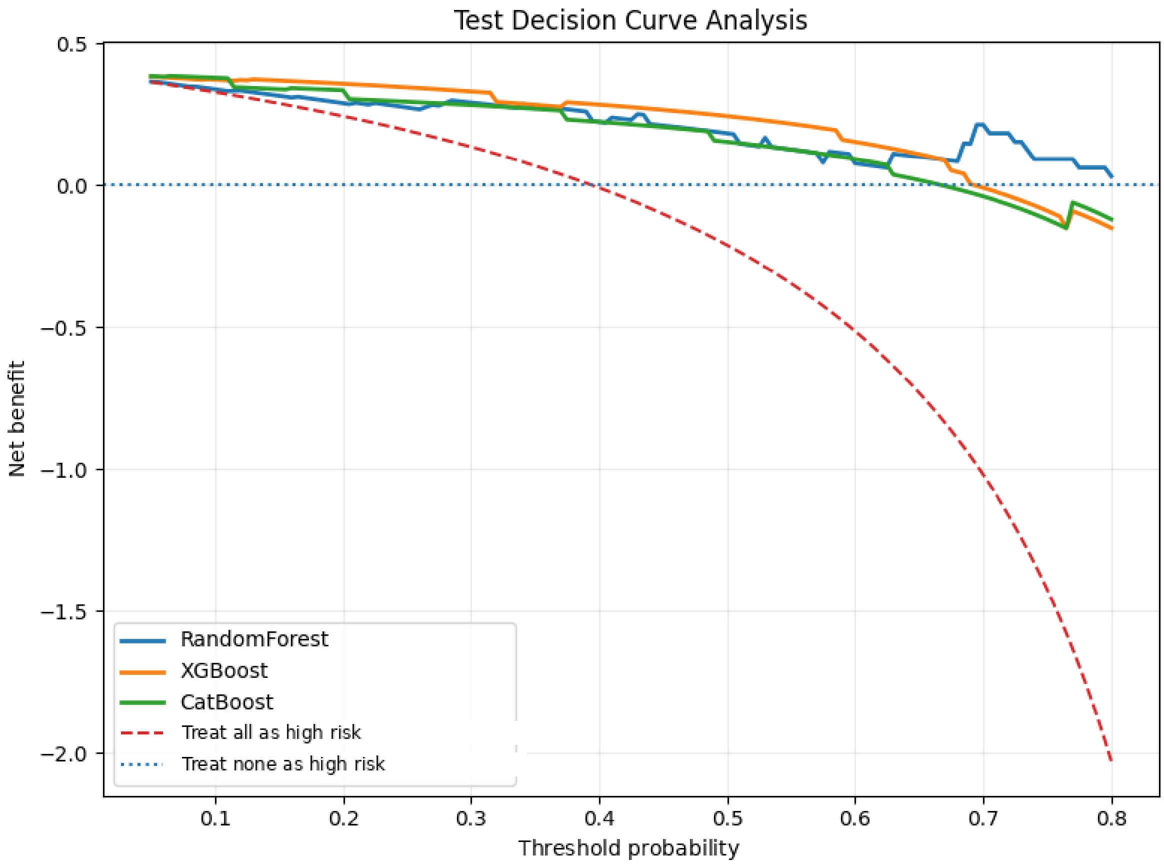

Figure S4. Decision curve analysis for the conventional 80/20 test set.

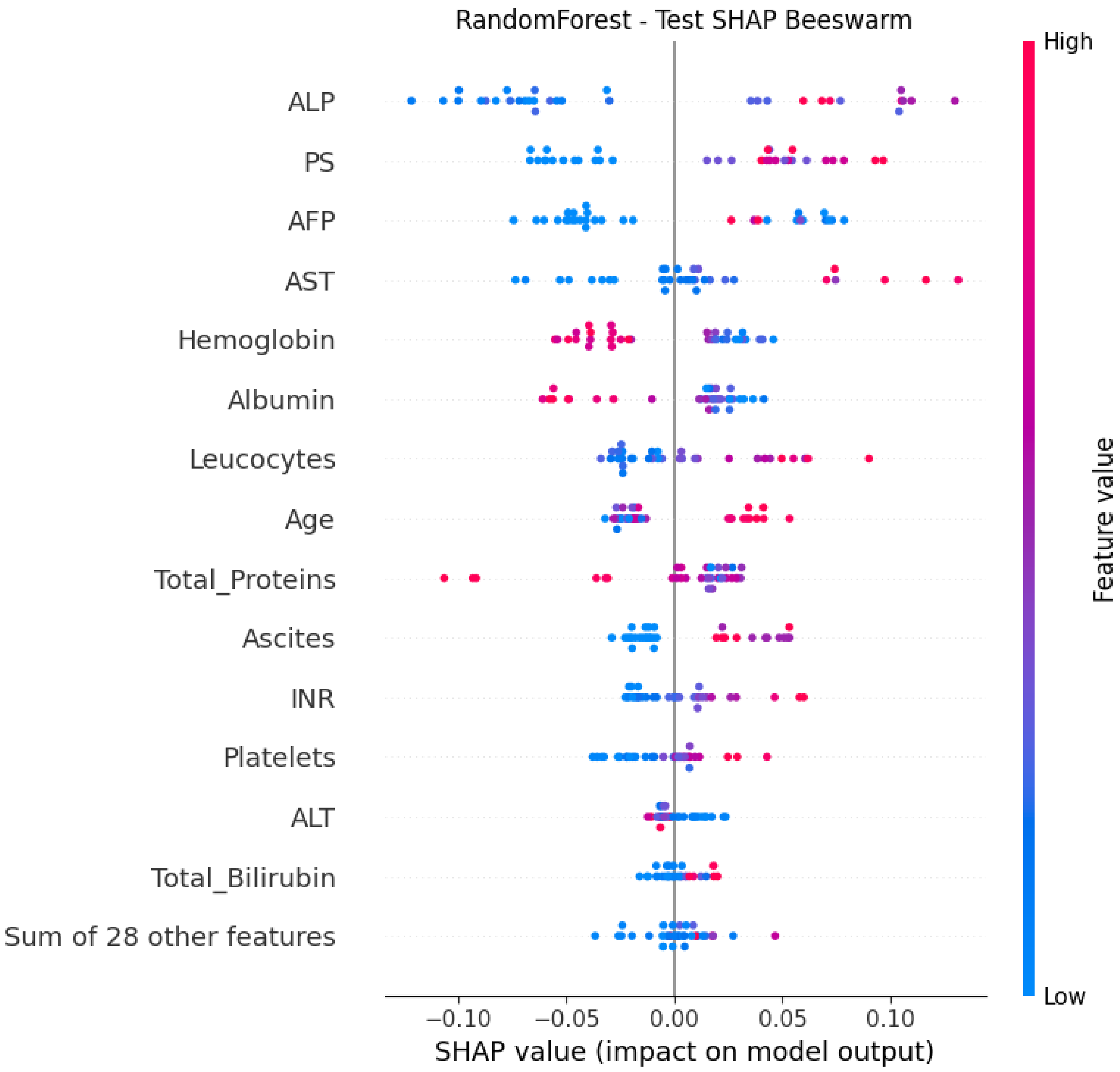

Figure S5. SHAP summary plot of the RF model for the conventional 80/20 test set.

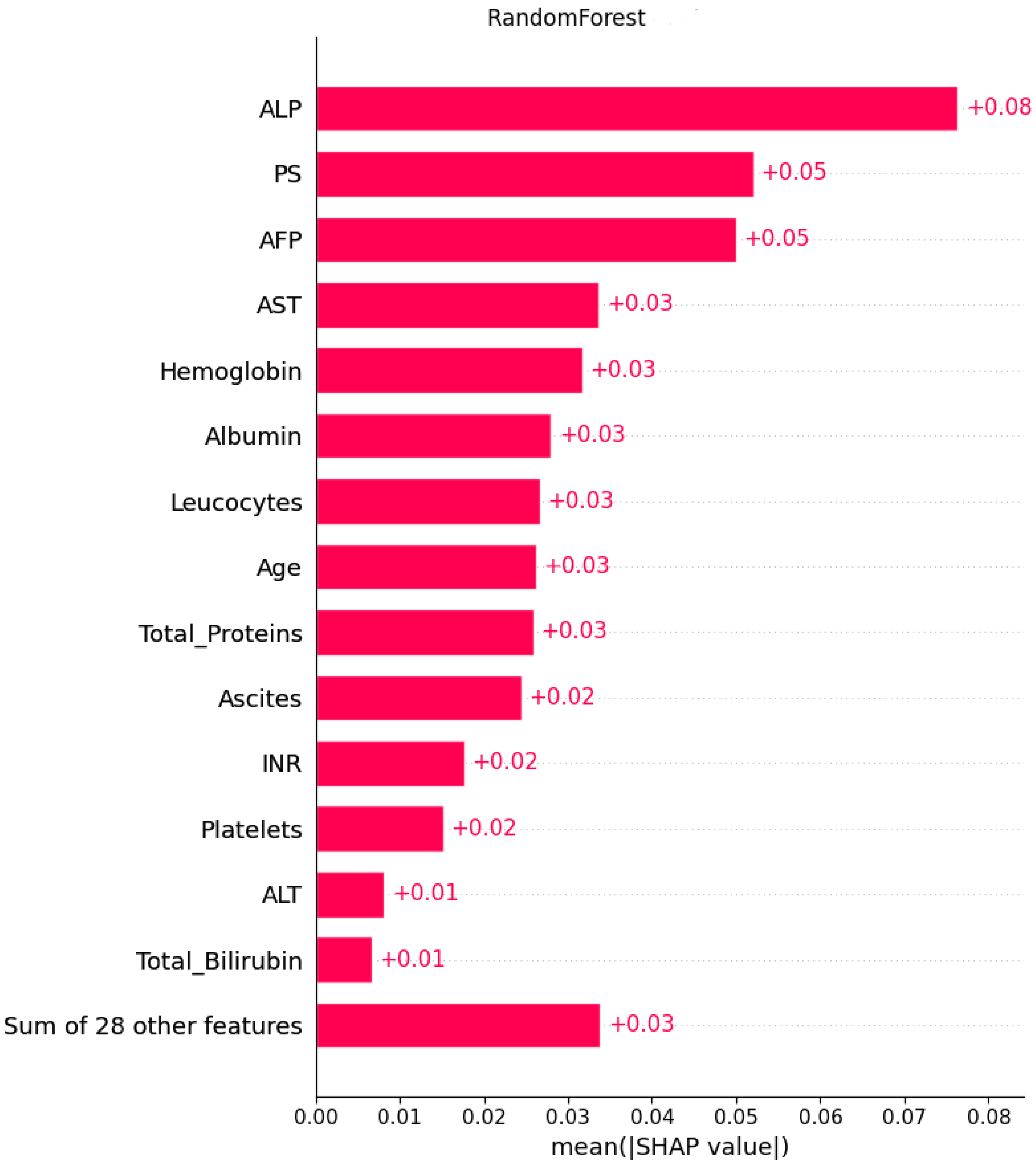

Figure S6. Global predictor importance based on mean absolute SHAP values of the RF model for the conventional 80/20 test set.
